# Understanding the success and failure of online political debate: Experimental evidence using large language models

**DOI:** 10.1126/sciadv.adv7864

**Published:** 2025-07-23

**Authors:** Tobias Heide-Jørgensen, Gregory Eady, Anne Rasmussen

**Affiliations:** ^1^ Department of Political Science, University of Copenhagen, Copenhagen 1353, Denmark.; ^2^ Department of Political Economy, King’s College London, London, UK.

## Abstract

Online political debate is frequently lamented for being toxic, partisan, and counterproductive. However, we know little about how core elements of political debate (justification, tone, willingness to compromise, and partisanship) affect its quality. Using text-based treatments experimentally manipulated with a large language model, we test how these elements causally affect the quality of open-text responses about issues important to the US and UK public. We find substantial evidence that differences in justification, tone, and willingness to compromise, but not partisanship, affect the quality of subsequent discourse. Combined, these elements increase the probability of high-quality responses by roughly 1.6 to 2 times and substantially increase openness to alternative viewpoints. Despite the ability to bring about substantial changes in discourse quality, we find no evidence of changes in political attitudes themselves. Our findings demonstrate how adapting approaches to online debate can foster healthy democratic interactions but have less influence on changing minds.

## Teaser

Adjusting tone, justification, and compromise improves online debate quality but does not change political attitudes.

## INTRODUCTION

Political discussion frequently takes place in online settings, particularly on social media. These platforms enable large and diverse segments of society to engage in politics and to discuss political issues at minimal cost (*1*–*3*). Early on, many were optimistic that this technology would facilitate political engagement centered around meaningful political exchanges and across political lines (*4*–*7*). Today, however, many concede that online political discourse has fallen short of the ideals of high-quality and open-minded debate. Online political discourse is often plagued by partisan polarization and hostility (*8*–*12*), incivility and intolerance (*13*, *14*), and the spread of low-quality and false information (*15*–*17*).

However, despite this, recent experimental research shows that when people with opposing partisan identities or issue positions discuss politics, in both offline and online settings, the consequences are beneficial (*18*–*23*). Political discussion is shown to induce compromise on political issues by moderating issue positions, decreasing affective polarization, increasing beliefs in the value of alternative perspectives, and increasing tolerance. These findings align with older work on the effects of participation in deliberative forums (*24*, *25*). However, outside of controlled experimental settings, research suggests that political intergroup contact can also short-circuit productive debate: Research finds that exposure to out-partisans on social media increases polarization on political issues (*9*), that out-partisan animosity drives social media engagement (*26*), and that political discussion among family members from opposing political parties can strain family ties (*27*).

Here, we study the nature of online political debate, which is often marked by brief exchanges of views and arguments between two or more individuals who disagree on a political issue. Research regarding social media interactions, for example, emphasizes their often short, one-shot (*28*–*30*), and many-to-many (*31*) nature. Here, we focus specifically on the quality of online political debate, defined by reasoned engagement, a willingness to compromise, and the avoidance of uncivil and partisan attacks. We argue that moving beyond the mixed evidence concerning the effects of and on political debate requires investigation of how differences in approaches to debate, observed in the very basic elements of discourse, affect its quality (*32*). In particular, while an increasing amount of work is dedicated to studying the effects of political debate and discussions at a general level, we lack a strong empirical understanding of its foundations at the microlevel. As a result, we do not fully understand why political debate online might be productive in some cases but not in others [but see (*33*, *34*)]. To remedy this, we draw on insights from the rich theoretical literature on deliberation and discourse quality (*35*–*38*). From this body of research, we identify three key attributes that should be linked to the quality of online political debate: (i) the use of evidence and justifications to support one’s claims, (ii) the use of a respectful tone, and (iii) an openness to compromise. Furthermore, given its central role in contemporary politics, we examine (iv) the role of partisanship in affecting discourse quality.

We expect these four core elements of discourse to matter for the quality of subsequent debate, and for political moderation more generally, for the following reasons. First, a primary element of high-quality debate is the ability of those involved to substantiate their political arguments with reference to evidence or otherwise reasoned justifications for them. Receiving justifications through “reason-giving” enables people to assess the validity of political claims and their implications (*38*). One can expect, therefore, that reason-giving fosters higher-quality online conversations (*39*, *40*), whereas the use of emotional rhetoric (*41*), often associated with moral outrage (*42*, *43*), may weaken it. Thus, although a well-known study shows that emotional argumentation is linked to higher engagement (*44*), by being perceived, often incorrectly, as moral outrage (*44*), it may lead to lower-quality political interactions.

Second, politics in general and online in particular has become increasingly uncivil (*45*), and research suggests that incivility (e.g., sarcasm, name-calling, mockery, etc.) or disrespectful tone more generally is detrimental to online and offline debate, although opinions differ [see (*46*, *47*)]. Conversely, being respectful during political disagreements can be expected to boost the quality of online debate, encouraging an interlocutor to be more willing to listen and to believe that they are engaging with someone arguing in good faith.

Third, signaling a willingness to hear the other side, to accept valid arguments even if they challenge one’s own position, and, ultimately, to signal openness to compromise are all likely to encourage high-quality online debate (*35*, *36*, *48*). Although, relatively, little is known about whether and how expressing an openness to compromise affects political debate in practice, related work suggests its potential importance (*23*, *33*, *49*).

Last, partisanship is often argued to be the most influential social identity affecting political behavior and a major factor in how people perceive and engage in discussions with out-partisans and in-partisans online (*50*, *51*). For example, research shows that social media content concerning political out-groups elicits more anger and engagement than content concerning in-groups (*26*, *52*), and people tend to discriminate against and harbor strong dislike toward out-partisans in general (*53*–*55*). That is, one can expect that political discussion with out-partisans will lower the quality of online debate than discussion with in-partisans. On the basis of the above, we test the following preregistered hypotheses (the order of the hypotheses differs slightly from that in the preregistration; see text S2):

Hypothesis 1 (argument quality): Using evidence to justify a political position, using a respectful tone, signaling openness to compromise, and being a copartisan will each increase the probability of receiving high-quality political responses during political debate.

Hypothesis 2 (mirroring): Responses will mirror in content and tone the elements of discourse used by one’s interlocutor. That is, evidence-based arguments will cause an increase in responses that are evidence-based and/or provide justifications for a position, disrespectful arguments will cause an increase in disrespectful responses, arguments signaling openness to compromise will cause an increase in responses also signaling openness to compromise, and out-partisan signaling will cause an increase in partisan attacks.

Hypothesis 3 (openness to other beliefs): Using evidence to justify a political position, using a respectful tone, signaling openness to compromise, and being a copartisan will increase a person’s openness to alternative viewpoints on an issue discussed.

Hypothesis 4 (mobilization): Using evidence to justify a political position, using a respectful tone, signaling openness to compromise, and being a copartisan will increase political engagement in terms of interest in learning more about arguments regarding an issue discussed.

Hypothesis 5 (polarization): Using evidence to justify a political position, using a respectful tone, signaling openness to compromise, and being a copartisan or nonpartisan will reduce ideological and affective polarization.

Hypothesis 6 (moderation): Using evidence to justify a political position, using a respectful tone, signaling openness to compromise, and being a copartisan will increase moderation in an individual’s position on an issue discussed.

To test the hypotheses, we use a large language model (LLM) in an experimental setup to systematically vary the core elements of online political debate in the form of counterarguments (*56*). We conduct preregistered survey experiments (see text S3) in the US and UK to examine how the basic elements of political discourse affect the overall quality and content of subsequent replies, as well as broader political beliefs and attitudes. Using an LLM allows us to experimentally manipulate counterarguments tailored to each respondent’s position on a political issue that is important to them and vary these arguments along four dimensions of deliberation: justification, tone, willingness to compromise, and partisan signaling. Using an LLM to construct treatments in this way provides a unique opportunity to develop high-quality and naturalistic counterarguments on the fly and test how these treatments independently affect respondents’ replies, which is something not possible in traditional survey research. Using rich text-based and closed-form survey outcomes, we comprehensively document the effects of a core set of elements of online political debate and investigate how they shape perceptions of an interlocutor.

Our results demonstrate that (i) differences in approaches to political discourse have substantial effects on the quality of resulting replies and (ii) the core elements of discourse complement each other in eliciting aspects of healthy discussion and generating positive spillover effects across dimensions. Combined, the use of evidence-based argumentation, signaling a willingness to compromise, using a respectful tone, and not signaling a partisan affiliation can double the probability of receiving a high-quality political response compared to scenarios where these elements are absent. Furthermore, we show that manipulating the core elements of online political debate not only fosters replies of a similar nature (e.g., evidence-based justifications, disrespectful tone, and openness to compromise each leading to responses of a similar nature) but also that their effects spill over in important ways to promote complementary aspects of debate. For example, signaling a willingness to compromise increases the use of evidence in a subsequent response, and using evidence-based arguments decreases responses that are disrespectful. By contrast, whether arguments are put forward by an in-partisan or out-partisan does not consistently affect the quality of subsequent replies.

Our results also shed light on how the elements of online political debate affect perceptions of the interlocutor, offering a plausible explanation for their impact on the overall quality of the debate. Empirically, we show that approaches to online political debate influence whether an interlocutor is seen as open-minded, constructive, well informed, respectful, and capable of making sound arguments. Furthermore, we demonstrate that how one approaches online political debate not only enhances the quality of responses received but also increases respondents’ openness to alternative viewpoints and shows potential to reduce affective and ideological polarization. Last, although we show that manipulating the elements of discourse has substantial effects on the quality of subsequent replies, we find no evidence that this affects changes in political attitudes.

Overall, our findings demonstrate that differences in approaches to political argumentation and discussion can have profound and substantial effects on the quality of online debate. Moreover, they reveal that positive elements of online political debate not only complement each another but also generate positive spillover effects across multiple dimensions. The ability of certain approaches to online debate to foster openness to alternative viewpoints and potentially encourage future discussion among out-partisans is particularly noteworthy. This underscores the potential for citizens to develop more high-quality interactions, even in our highly polarized and partisan political climate. Our research also highlights the critical role of learning how to formulate arguments that improve perceptions of oneself as an interlocutor, a key factor in determining the effectiveness of quality argumentation. The results also show, however, that the ability to affect the quality of subsequent replies does not necessarily come with meaningful changes in political attitudes. Thus, although there is a potential to substantially improve discourse quality, an important outcome in itself, our results highlight the limitations of improving online political debate quality when it comes to persuasion or ideological moderation.

## RESULTS

As outlined in Materials and Methods, we examine the effects of the features of conversation on the quality of political debate using preregistered survey experiments in the UK and the US. We first ask participants to record, in an open-text field, a political position that they hold and their reasons for holding it. An LLM then generates counterarguments to that position in accordance with randomly assigned treatment conditions. As outcomes, we ask research subjects to respond in an open-text field to the counterargument and to complete a battery of closed-form survey questions (see Materials and Methods for details).

Below, we present the results using data pooled from both the US and UK samples. We also provide analogous results for each country separately. However, because of space constraints and their high degree of similarity, detailed discussions of these country-specific results are included in text S6. For all outcomes, we estimate the treatment effects using ordinary least squares (OLS) regressions that include the four treatment indicators as predictors in each model (we provide regression tables for each figure in text S10). To address potential issues of multiple comparisons, we provide adjusted *P* values (*P*_adj._) to control for the false discovery rate, thus accounting for the expected proportion of falsely rejected null hypotheses (*57*).

### Discourse quality

**Fig. 1.**
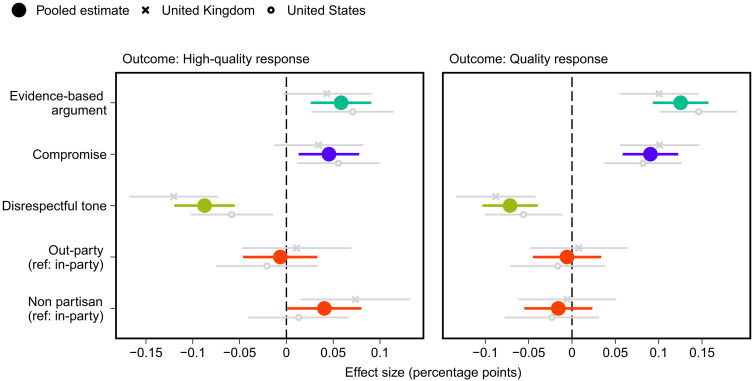
Effects of attributes of counterargument on probability of high-quality and quality reply. Estimated treatment effects with 95% confidence intervals from OLS regression models that include all treatment variables. Ref, reference category. *N*_pooled_ = 3303, *N*_UK_ = 1537, and *N*_US_ = 1766.

How do the features of conversation affect the overall quality of online political debate? To test this, Fig. 1 presents the estimated effects of each treatment on the probability that a respondent replies with a “high-quality” open-text response (qualified justification or willingness to compromise plus respectful and no partisan attack) or a “quality” response (any justification or willingness to compromise plus respectful and no partisan attack). Consistent with hypothesis 1, counterarguments that use evidence-based justifications, signal a willingness to compromise, and are respectful all independently increase the probability of high-quality or quality responses (for all, *P* < 0.01, *P*_adj._ < 0.01). Responding with an evidence-based counterargument (relative to an emotion-based response) increases the probability of eliciting a high-quality response by six percentage points, indicating willingness to compromise by five percentage points, and being respectful by nine percentage points. Indicating that one belongs to the out-party or in-party reduces the probability of eliciting a high-quality response, compared to no partisan signaling (*P* < 0.05, *P*_adj._ < 0.1). The effect of the nonpartisan (no party mentioned) treatment has a significant positive effect when compared separately to both the out-party (*P* < 0.05, *P*_adj._ < 0.05) and the in-party (*P* < 0.05, *P*_adj._ < 0.05) treatment conditions. Put differently, respondents are more likely to respond with high-quality replies to nonpartisans even compared to those from their own preferred party. One might wonder whether the relatively weak effects of partisanship are due to the treatment being too subtle. Two findings strongly suggest that this is not the case. As shown further below, interlocutors are more likely to receive partisan attacks when they signal out-partisanship, and in-partisans are far less likely to be perceived as ideologically extreme compared to out-partisans. Refraining from partisan signaling increases the probability of high-quality replies by four to five percentage points. This result does not hold, however, for the quality measure (right panel of Fig. 1), which reflects a lower standard for what constitutes a reply with a less sophisticated justification. A test for differential effects across all treatments between the UK and US samples reveals no significant differences in treatment effects between the two countries (see text S6 for details).

To put the magnitude of these effects in perspective, we calculate the change in probability of receiving a high-quality response if a counterargument is evidence-based, is respectful, signals a willingness to compromise, and is nonpartisan, as compared to the alternative conditions. On average, the magnitude of the effect of this type of reply is substantial, increasing the probability of a high-quality reply by roughly two times (24% versus 47%). Because it is possible that interactions between treatments may affect this estimate, we also conduct a likelihood ratio test to compare a saturated model that includes a full set of interactions to a model without them. The inclusion of interactions does not significantly improve model fit (*P* = 0.58). Nevertheless, we can examine the difference in high-quality responses from the subset of respondents who received the exact combination of “best” treatment conditions (*n* = 151) (i.e., evidence-based, respectful, open to compromise, and nonpartisan) and compare these to the subset respondents who received the alternative combination of treatment conditions (*n* = 148). Comparing these groups results in a similar, albeit noisy, estimate (29% versus 47%), a roughly 1.6 times increase in high-quality responses.

**Fig. 2.**
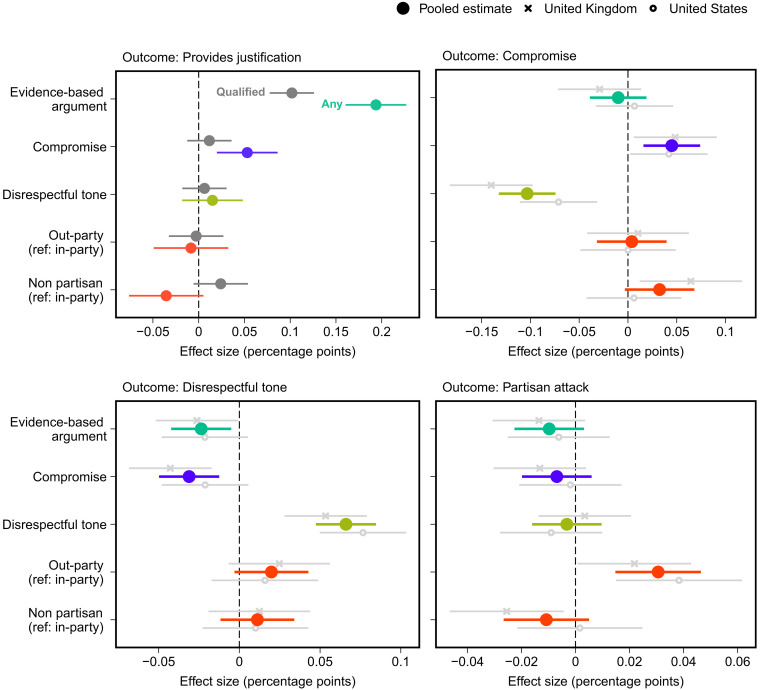
Effects of attributes of counterargument on detailed discourse-level outcomes of the reply. Estimated treatment effects with 95% confidence intervals from OLS regression models that include all treatment variables. A reply is coded as providing a qualified justification if it makes one or more clear linkages between a position and a reason for taking that position. Any justification includes inferior justifications that do not make these linkages between a position and a reason [see (*78*, *79*)]. Ref, reference category. *N*_pooled_ = 3303, *N*_UK_ = 1537, and *N*_US_ = 1766.

We now test the effects of the treatments on each discursive element of respondents’ replies. We recall that we expect that the quality and content of responses to counterarguments will mirror that of the counterargument (hypothesis 2)—that, in political argumentation, one gets what one gives. As the results presented in Fig. 2 show, we find that this is generally the case: Evidence-based arguments (relative to emotion-based responses) increase the probability of replies that provide justifications for a position (*P* < 0.01, *P*_adj._ < 0.01), signaling a willingness to compromise increases respondents’ own willingness to compromise (*P* < 0.01, *P*_adj._ < 0.01), a disrespectful tone increases the probability of a disrespectful reply (*P* < 0.01, *P*_adj._ < 0.01), and indicating that one is an out-partisan increases the probability of a partisan attack (*P* < 0.01, *P*_adj._ < 0.01). In fig. S20 in text S9, we show results when the outcome is any mention of partisanship at all (i.e., not only partisan attacks). Results show that evidence-based arguments, signaling a willingness to compromise, and not signaling one’s partisanship decrease any mention of partisanship in respondent’s replies.

That one gets what one gives in political argumentation is important to document experimentally. However, the effects of the treatments do not only affect the analogous element in a respondent’s reply. They also provide meaningful complements to each other. As Fig. 2 shows, for example, in addition to evidence-based arguments (compared to emotion-based responses) increasing respondents’ justifications of their issue positions, using evidence also decreases disrespectful rhetoric (*P* < 0.05, *P*_adj._ < 0.05), and compromise increases the probability that respondents provide a minimal justification for their issue positions (*P* < 0.01, *P*_adj._ < 0.01). In addition, signaling one’s openness to compromise decreases respondents’ use of disrespectful language (*P* < 0.01, *P*_adj._ < 0.01). The effects of the salutary elements of discourse, in other words, are complementary across the different elements of debate that constitute high-quality political discussion.

Last, one might expect that the use of a disrespectful tone in political discourse would undermine the positive effects of other positive elements. For instance, once individuals are subjected to ridicule or sarcasm, they may become unwilling to engage in meaningful online political debate, regardless of the substance of the argument. However, in an exploratory analysis presented in text S9, we find no evidence to support this. Even when counterarguments are presented disrespectfully, the effects of evidence-based argumentation (compared to emotion-based responses) and signaling a willingness to compromise remain similar in magnitude to when counterarguments are delivered respectfully. Even in toxic environments, in other words, the potential benefits of appealing to evidence and compromise persist.

### Openness to alternative perspectives and further information

**Fig. 3.**
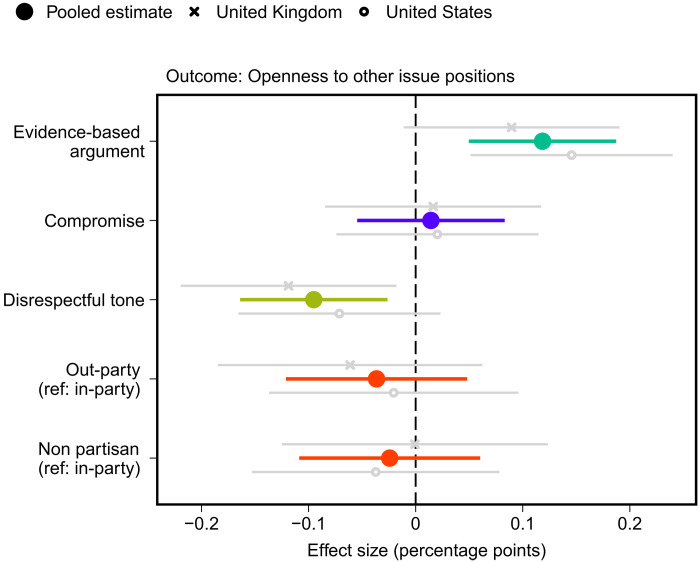
Effects of attributes of counterargument on openness to other beliefs. Estimated treatment effects with 95% confidence intervals from OLS regression model that includes all treatment variables. Openness to other beliefs is an index based on three survey items (see text S3.1). Ref, reference category. *N*_pooled_ = 3231, *N*_UK_ = 1510, and *N*_US_ = 1721.

Next, we test whether the different elements of debate affect respondents’ openness to alternative perspectives on the issue most important to them and their willingness to learn more about arguments concerning the issue. Figure 3 presents results for openness to alternative perspectives. Consistent with hypothesis 3, the results show that providing evidence-based counterarguments (compared to emotion-based responses) (*P* < 0.01, *P*_adj._ < 0.01) and using a respectful tone (*P* < 0.01, *P*_adj._ < 0.05) cause increases in respondents’ openness to alternative viewpoints on the issue at hand. However, whether the argument comes from a copartisan or out-partisan has no significant effect nor does signaling a willingness to compromise.

We also evaluate whether the elements of online political debate mobilize people to seek further information about the issue (hypothesis 4). We recall that we measure this outcome by whether respondents click a link to access more information about the issue. Although many respondents indicate interest in learning more (50% click the link), none of the treatments significantly affect this quasi-behavioral measure of willingness to learn more (see fig. S1 in text S4).

### Political orientations

**Fig. 4.**
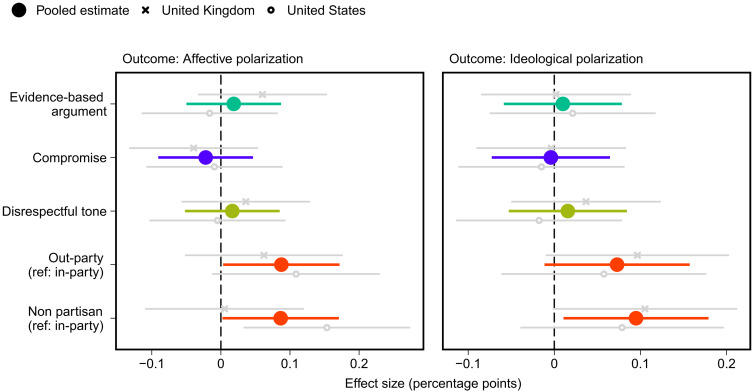
Effects of attributes of counterargument on affective and ideological polarization. Estimated treatment effects with 95% confidence intervals from OLS regression models that include all treatment variables. Affective polarization is measured as the absolute difference in feelings toward the two major parties. Ideological polarization is an index based on answers to 10 economic and cultural issues (for question details, see text S3.1). Ref, reference category. Affective polarization sample: *N*_pooled_ = 3275, *N*_UK_ = 1528, and *N*_US_ = 1747. Ideological polarization sample: *N*_pooled_ = 3271, *N*_UK_ = 1528, and *N*_US_ = 1743.

Last, we document whether the different elements of debate affect political orientations, both in terms of ideological and affective polarization and moderation on the issue discussed. As shown in Fig. 4, when a counterargument is given by a copartisan (the baseline category), both affective polarization (*P* < 0.05; *P*_adj._ < 0.1) and ideological polarization (*P* < 0.05; *P*_adj._ < 0.1) are lower than when given by an out-partisan or nonpartisan (consistent with hypothesis 5). However, we observe no other effects on polarization.

**Fig. 5.**
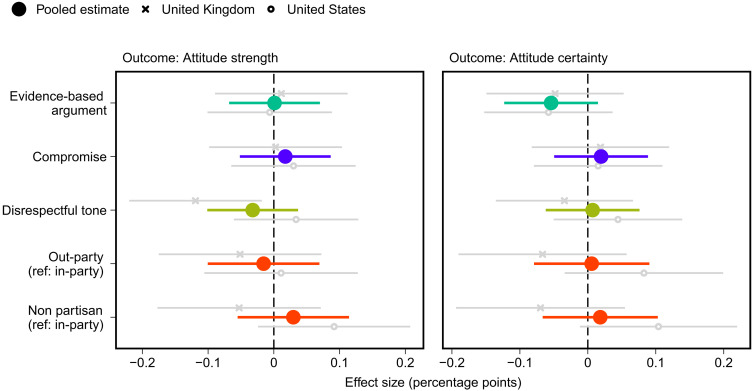
Effects of attributes of counterargument on attitude strength and attitude certainty regarding the issue. Estimated treatment effects with 95% confidence intervals from OLS regression models that include all treatment variables. Ref, reference category. Attitude strength sample: *N*_pooled_ = 3230, *N*_UK_ = 1510, and *N*_US_ = 1720. Attitude certainty sample: *N*_pooled_ = 3231, *N*_UK_ = 1510, and *N*_US_ = 1721.

Do the different elements of political debate influence whether respondents moderate their position on the issue? We recall that we expect that counterarguments that are evidence-based (rather than emotion based), are respectful, signal a willingness to compromise, or come from copartisans will moderate people’s issue positions and reduce certainty about issue positioning (hypothesis 6). As shown in Fig. 5, no treatment significantly affects either issue positioning or respondents’ certainty about their position on the issue. Thus, although we find that elements of high-quality counterarguments substantially improve discourse quality (see Fig. 1), we find no evidence that they are more likely to persuade, a result that is consistent with related recent work (*58*, *59*).

### Effects on perceptions of an interlocutor

Above, we presented evidence that using evidence-based arguments, being respectful, and signaling a willingness to compromise substantially increase the probability of high-quality responses. These effects can likely be attributed to how the content and tone of political discourse influence participants’ assessments of the qualities of the person with whom they are conversing (*60*, *61*) and, thus, the utility of putting effort into subsequent debate. To investigate this, we document in an exploratory analysis the effects of each treatment on respondents’ perceptions of their interlocutor. We examine the effects of the treatments on six perceptions: whether a respondent believes that their interlocutor (i) makes good arguments (for simplicity of presentation, we measure perceptions of whether the social media user makes “good arguments” by summing respondents’ perceptions of whether their arguments are “strong” and whether they are “reasonable”; effects on these two component measures separately are presented in text S8), (ii) is open to changing their mind, (iii) is engaging in constructive dialogue, (iv) is well informed about politics, (v) is disrespectful, and (vi) is ideologically extreme.

**Fig. 6.**
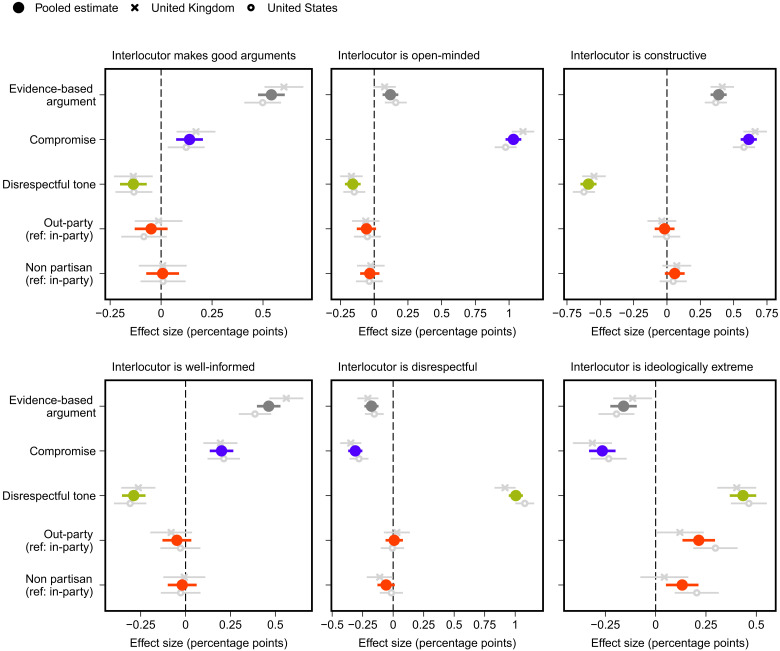
Effects of attributes of counterargument on perceptions of the interlocutor. Estimated treatment effects with 95% confidence intervals from OLS regression models that include all treatment variables. Ref, reference category. *N*_pooled_ = 3280 (average), *N*_UK_ = 1529 (every outcome), and *N*_US_ = 1751 (average).

As the results presented in Fig. 6 show, the use of evidence-based arguments (versus emotion-based responses) and signaling an openness to compromise cause respondents to see interlocutors as making good arguments, being open-minded, being constructive, being better informed about politics, and being less disrespectful and ideologically extreme (for all coefficients, *P* < 0.01, *P*_adj._ < 0.01). Given the outcomes, the use of evidence and compromise are important complements. Signaling willingness to compromise, for example, increases perceptions of the quality of an interlocutor’s argument and whether the interlocutor is well informed. This is the case although it does not, for instance, affect the substance of the argument itself. Analogously, providing evidence-based arguments increases perceptions of their being open-minded and decreases perceptions of an interlocutor as disrespectful.

It is expected that using a disrespectful tone decreases perceptions of an interlocutor as constructive or open-minded (as shown in Fig. 6). However, disrespectful tone also affects how participants assess the strength of an interlocutor’s argument and decreases beliefs about whether an interlocutor is well informed (for both, *P* < 0.01, *P*_adj._ < 0.01). That is, regardless of whether the content of an argument is emotion-based or evidence-based, using disrespectful language nevertheless negatively affects how one assesses the quality of that argument and whether the person being spoken with is knowledgeable about the issue at hand. Disrespectful language also causes increases in perceptions of an interlocutor as ideologically extreme and decreases perceptions that they are open-minded or constructive (for both, *P* < 0.01, *P*_adj._ < 0.01).

In addition, we find that the interlocutor’s partisanship affects how ideologically extreme respondents perceive them to be (*P* < 0.01, *P*_adj._ < 0.01). This supports recent research that shows that once a policy position of an in-partisan or out-partisan is known, the value of partisanship as a heuristic for beliefs about that individual is relatively minimal (*62*–*64*). We find no significant evidence that partisanship affects perceptions otherwise.

Last, in fig. S2 in text S5, we show that perceptions of an interlocutor predict whether participants provide high-quality responses: Perceiving one’s interlocutor as giving good arguments and being open-minded increases the probability of a high-quality reply (for both, *P* < 0.01, *P*_adj._ < 0.01), and perceiving an interlocutor as disrespectful decreases it (*P* < 0.05, *P*_adj._ < 0.05). Combined with the findings in Fig. 6, this suggests that beliefs and assumptions about an interlocutor may be an important potential reason why the content and tone of political discourse affect the quality of resulting online political debate.

### Auxiliary analyses

We conduct a number of exploratory analyses to investigate heterogeneity in the effects of each treatment on the quality of respondents’ open-text responses. As reported in text S9, we test whether the treatment effects vary by respondents’ gender (fig. S26), age (fig. S27), education (fig. S28), social media use (fig. S29), partisanship (fig. S23), and ideology (fig. S24) and whether respondents are on the ideological extremes (fig. S25). We also tested whether treatment effects varied if we divided issues into overall categories of economic, cultural, and other issues, finding little evidence of this (see fig. S30). Overall, we find very few differences in effect sizes by subgroup or treatment status, a set of null findings that are consistent with growing evidence that shows that effect homogeneity, not heterogeneity, is often the norm (*58*, *65*, *66*).

## DISCUSSION

Online political debate among ordinary citizens would ideally be founded in good-faith argumentation and result in meaningful exchanges about important political issues (*4*–*7*). Instead, online political debate is often lamented for being toxic, partisan, and polarized, crowding out otherwise sober deliberation (*8*–*12*). However, although recent research has sought to examine the causal effects of whether one engages in debate and with whom (*18*–*23*), we know relatively little about whether and how the microlevel features of online political debate affect the quality of political exchange.

This article addresses this by building on recent innovations in LLMs to provide an empirical foundation for our understanding of the effects of the core elements of online political debate on discourse quality, attitudes, beliefs, and perceptions. We demonstrate that using evidence-based justifications (rather than emotion-based ones), using a respectful tone, and signaling a willingness to compromise produce exceptionally large effects on the quality of responses. Our findings also indicate that the nature of one’s approach to debate is likely to result in responses of a similar kind: Evidence-based arguments prompt evidence-based replies, respectful language fosters respectful replies, and signaling openness to compromise encourages similar openness from interlocutors.

The elements of online political debate also generate positive spillover effects above and beyond eliciting responses of a similar kind. For example, expressing an openness to compromise increases the probability of responses that contain justifications for political positions, and using evidence-based arguments and signaling a willingness to compromise decrease disrespectful replies. The results show that engaging people with evidence and using a respectful tone not only have direct effects on the contents of discourse itself but also broaden individuals’ openness to alternative political viewpoints. Furthermore, our research demonstrates how core elements of online political debate affect perceptions of an interlocutor. Because political discussion is costly in terms of time and cognitive effort, beliefs about an interlocutor can affect the amount of effort invested in debates online. Last, our results show that improving the quality of online political debate does not necessarily translate into changes in attitudes concerning the issue discussed. We find no evidence that evidence-based argumentation, tone, openness to compromise, nor differences in partisanship affect the strength or certainty of people’s issue positions.

Our results carry important implications for our understanding of online political conversations. The relatively weak effects of partisanship suggest that, even in polarized systems and on highly contentious issues, adjusting one’s approach to debate can substantially improve the quality of political responses. Consistent with recent research, this suggests that partisan identity and in-group/out-group dynamics may lose importance when people know the issue positions and arguments of the people with whom they are interacting (*62*–*64*) [however, also see (*67*)]. It also suggests that counterproductive online political debate may have more to do with approaches to debate than the partisan identities of those involved. From a normative perspective, it is encouraging to observe that content and tone can potentially outweigh potential animosities based on partisanship or perhaps other forms of identity. Our results suggest that adopting certain approaches to talking to opponents can foster mutual understanding and respect, if not overcoming political divides. In this way, they underscore the potential for citizens to learn how to develop higher-quality interactions and the potential to use inputs from LLMs to guide discussion in online political forums.

Our results nevertheless demonstrate important limitations to using more constructive approaches to online political debate. Specifically, we show that high-quality discourse is no more persuasive than low-quality discourse, a result that runs counter to the expectations in deliberative theory [e.g., (*36*, *68*)] but aligns with recent work on attitude formation (*58*, *59*). Thus, while arguments are generally effective at influencing people’s beliefs, as research has shown [see, for example, (*58*)], the discursive presentation or quality of the argument does not appear to play a significant role in this process. One explanation for this is that the participants chose the issues discussed themselves, which may account for their particularly strong and resistant opinions. It is possible that discussing issues that are less deeply felt might result in greater attitude change (*69*–*71*). However, the issues discussed most intensely online are likely those that people are most personally invested in. Nevertheless, even if higher-quality counterarguments are not more effective at changing minds, the fact that positive elements of discourse promote overall higher-quality online political debate and larger openness to alternative perspectives is valuable in itself. This underscores the potential for fostering more harmonious and open-minded debate between individuals with deeply divergent views on political issues.

While this article documents experimentally a broad range of basic facts about how the microlevel features of online political debate affect the quality of political discussion, there remains substantial scope for future research into how approaches to debate shape political interactions. One promising avenue is to examine the downstream effects of high-quality discourse. This paper focuses on the effects of approaches to discourse on the quality of online political debate and related outcomes within a single interaction. This leaves room for examining whether the effects of longer-term or repeated interactions are stronger or otherwise different. For instance, high- and low-quality approaches to debate might have a contagious effect, leading to virtuous or vicious cycles in political discussion. Moreover, despite the substantial effects observed in our experiments, more frequent interactions may also yield even stronger effects, resulting in lasting changes in approaches to debate or political attitudes. In addition, future research should examine how the elements of political debate affect polarization toward people in general who hold an opposing view on an issue. Future research should also build on the insights of our study and investigate the microlevel features of online political debate in real-world settings further, a methodologically challenging but important avenue for research.

Empirically, we examine differences in the effects of evidence-based argumentation as compared to emotion-based responses, a specific type of nonevidence-based response that is both meaningful and common in online political discourse (*44*). However, other forms of nonevidence-based responses [e.g., appeals to fairness or to moral standards (*72*)] may result in different effects on the quality of discourse. We encourage future research to extend our findings by comparing a broad range of counterarguments and response strategies, including those grounded in, for instance, moral reasoning or fairness considerations.

Furthermore, differences in the effects of approaches to discourse may also vary across offline and online contexts. In online debate, for example, individuals may become habituated to the rapid, informal communication characteristic of social media, which could dampen their sensitivity to variations in argument quality. By contrast, offline debates—often governed by more formal social norms—may elicit stronger responses to differences in discourse, as interlocutors are less accustomed to the dynamic and occasionally fragmented nature of online discussion. Empirically, our analysis (see fig. S30) indicates that while treatment effects are broadly similar regardless of social media usage frequency, the magnitude of these effects are larger for respondents who use social media infrequently or not at all (significantly so for compromise and nonpartisanship). This finding underscores the importance of accounting for contextual and habitual factors when assessing the impact of discourse quality across different communication environments.

Last, it is crucial to also study the effects of different approaches to online political debate in less polarized countries than the US and UK. In less polarized settings, the baseline quality of political discussion may be higher, potentially leading to smaller observed effects. Conversely, the public in these settings might be more receptive to good-faith argumentation, potentially resulting in larger effects. This remains an important question for future research. Moreover, while our study finds primarily weak effects of partisanship on discourse quality, other identities may play important roles, varying in importance across political contexts or issues. For instance, the impact of an approach to argumentation may depend on whether an issue directly affects an identity group (e.g., women discussing reproductive rights, persons of color discussing affirmative action policies, and immigrants discussing immigration policies). Research into online political debate and dialogue, more broadly, would thus benefit greatly from examining how the effects of approaches to debate differ depending on the groups with which interlocutors identify and the topics under investigation. Our study provides an important foundation for this research by scrutinizing the dynamics of the microlevel features of online political debate and demonstrating how recent advancements in LLMs can serve as valuable tools in such an endeavor.

## MATERIALS AND METHODS

Testing the effects of approaches to political discourse on the quality of online political debate at the microlevel is methodologically challenging. On one hand, with observational data, people who select the type of political discussion that exposes them to high-quality counterarguments will be systematically different from people who select discussions with lower-quality arguments, making causal inference difficult. On the other hand, with experimental approaches that address the problem of causal inference, the general public will be interested in or have knowledge about only a small number of political issues, meaning that using experiments with fixed text-based treatments about, say, a researcher-chosen topic will be unlikely to elicit meaningful or realistic responses.

To overcome these challenges, we use an LLM to create experimentally manipulated counterarguments on the fly in response to open-text responses from respondents concerning the issue most important to them and the reasoning that they give for their position on the issue (*56*, *73*). The experimental protocol was reviewed and approved by the Institutional Review Board at the University of Copenhagen (see text S1 for details on ethical considerations). Using an LLM for this purpose offers two important advantages over conventional approaches. First, it allows us to independently combine discursive treatments into a coherent and naturalistic counterargument that resembles how people communicate online. Recent research shows that LLMs can produce arguments that are at least as convincing and original as those made by humans (*56*, *74*). Second, the content and tone of the counterarguments can be crafted to match the specific issue and issue position of any given respondent. This means that we do not, for instance, need respondents to select among a predefined list of issues that they may know or care little about. Using an LLM embedded within the design allows respondents to discuss issues that matter to them and respond to a counterargument tailored to those issues. As we show in text S3.3, although some issues occur more frequently than others (e.g., abortion, healthcare, and immigration), there is a large variation in the issues respondents consider most important. The details of our implementation of the LLM in our experimental setup are described further below.

### Data

We conduct survey experiments in the US (*n* = 1789) and the UK (*n* = 1551), countries that are important contexts for both theoretical and practical reasons. First, both countries have high levels of political polarization, and commentators frequently bemoan the state of their political discourse. Second, the LLM we use in our design, ChatGPT-4 (*75*), is primarily trained on English-language data, and research on counterarguments from these LLMs has been validated with English-language issues (*56*, *74*). Last, because the US and the UK are effectively two-party systems, signaling in-party and out-party identity for a partisanship treatment condition is straightforward.

Our sample consists of citizens in the US and the UK who identify with one of the major parties: Republicans or Democrats in the US and Conservative or Labour supporters in the UK. The data were collected between 17 July and 27 August 2023 through Prolific, an online provider shown to be of high quality compared to similar platforms (*76*, *77*). A total of 51 respondents (1%) were excluded because they failed an attention check, and 437 respondents (12%) were excluded either because they did not provide an intelligible issue position or because the LLM, for technical reasons, did not produce an output. As we note in the preregistration, our target sample of 3200 respondents can detect effects of at least five percentage points at 80% power and an effect of at least six percentage points at 90% power. Completion rate was 91% in the UK and 89% in the US. The samples were collected with a gender quota (equal number of men and women) and sought to result in an equal number of respondents in each of the four partisan groups.

Similar to other nonprobability samples, our sample does not exactly match the US and UK populations. Compared to the same party groups in the recent American National Election Study and British Election Study, our respondents are generally younger and slightly more male (see text S3.6). Research shows that heterogeneity in treatment effects in survey experiments across samples is small, suggesting that samples that are not wholly representative will nevertheless yield results similar to those from truly representative samples (*65*, *66*). Consistent with this and as discussed below and shown in figs. S21 to S30 in text S9, we find that the results do not vary strongly across various subgroups. In text S3.8, we also show that treatment assignment does not affect rates of survey attrition.

### Using an LLM to experimentally manipulate counterarguments

We test our hypotheses in a survey using a 2 × 2 × 2 × 3 full-factorial experimental design. The experiment is designed to mimic a political conversation in an online social media setting. Specifically, after completing a series of questions measuring pretreatment covariates, respondents were asked to imagine writing a social media post about a political issue that matters to them and then to write in an open-text field their position on that issue and why. Respondents were then presented with a counterargument from a fictitious social media user, where the counterargument was tailored, on the fly, to their issue position and reasoning by an LLM.

**Fig. 7.**
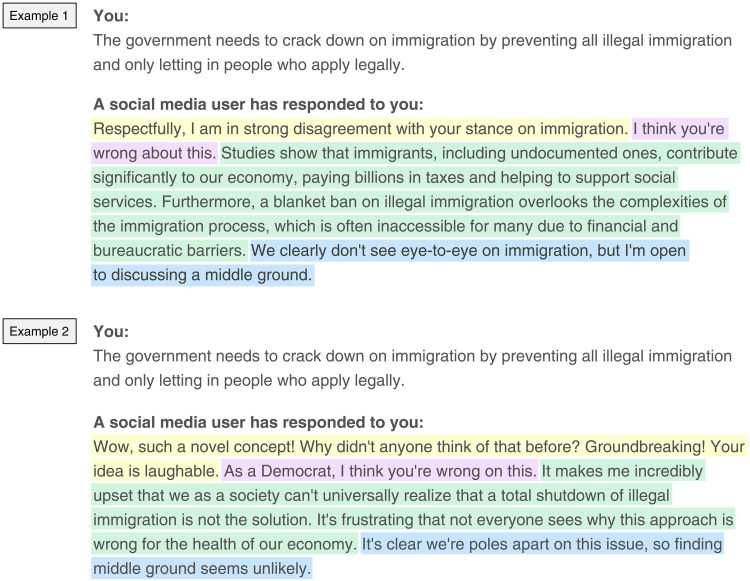
Example of experimentally manipulated responses to a survey respondent’s issue position from the LLM. This figure presents two hypothetical examples of the potential output that a respondent would see in response to their issue position. Highlighted text indicates the LLM-generated text relevant to each treatment condition: respectful/disrespectful treatment in yellow, in-partisan/out-partisan/nonpartisan in purple, evidence-based/emotional response in green, and compromise/uncompromising in blue. Treatment conditions presented in example 1 are respectful/nonpartisan/evidence-based argument/compromise. Treatment conditions presented in example 2 are disrespectful/partisan/emotional argument/uncompromising.

To experimentally manipulate the elements of each counterargument, we created LLM prompts for each of the treatment conditions. The one to two sentences generated for each of the four treatments were used to form a complete social media reply. We used separate prompts for each condition to avoid each treatment affecting each other (e.g., evidence-based arguments being different when paired with a respectful tone), which could lead to compound treatments. We tested and refined these prompts to ensure that they worked as intended, including conducting a pilot study in the two countries. Each of the four treatments in the 2 × 2 × 2 × 3 design were randomly assigned to respondents. As we show in text S3.7, randomization was successful, as treatment groups are well balanced on multiple covariates. The text of the counterargument was created by concatenating the text from the four separate calls (one per treatment) to the LLM. To see this by example, Fig. 7 shows an issue position from a hypothetical respondent and two hypothetical counterarguments generated by the LLM, where we highlight each treatment condition for reference. We detail the treatment conditions themselves below (for details on the prompts and how we used the LLM, see text S3.2).

The first part of each response experimentally manipulated tone. Respondents in the respectful condition read text that expressed strong disagreement with their position on the issue but did so in an explicitly respectful tone (e.g., using phrases such as “respectfully” or “with respect”). By contrast, respondents randomized to the disrespectful condition received a response that used sarcasm or otherwise ridiculed their issue position (using phrases such as “Oh brilliant!” or “Utter nonsense”).

The second element of the response signaled partisanship. We randomly varied whether the interlocutor of a respondent was presented as a member of the in-party or out-party or did not use any partisan signaling. This was done by adding “As a [Republican, Democrat]” (in the US) or “As a [Conservative, Labour supporter]” (in the UK) to a sentence that states that the respondent is wrong about the issue. Under the neutral (nonpartisan) condition, the sentence was the same but did not include a party affiliation.

Third, we manipulated the type of justification used to oppose the respondent’s issue position. In the evidence-based condition, the counterargument used factual or scientific evidence to rebut or criticize the respondent’s position. In the emotion-based condition, the interlocutor of a respondent opposed the respondent’s issue position by expressing, for example, being upset, angry, frustrated, annoyed, or sad that people believe what the respondent believes, without providing an argument for why they disagree. We note that we purposefully opt not to include a truly “neutral” condition (e.g., providing no counterargument or a minimal response such as “thank you” or “I disagree”). These brief counterreplies would not provide sufficient content for respondents to engage with meaningfully, nor would they be directly comparable to the more substantial responses present in the other treatment conditions. Although emotional responses occur frequently on social media (*44*) and, thus, are a natural comparison, the evidence-based condition could have been contrasted with other types of responses [e.g., fairness considerations (*72*)], which we will discuss at the end of the paper.

Last, we manipulated whether the interlocutor of a respondent expressed a willingness to compromise on the issue. This was done by stating that, while there is clear disagreement in opinions, the interlocutor is open to further discussion about the issue. An unwillingness to compromise was signaled, by contrast, by stating that their disagreement means that finding compromise is unlikely.

Similar to (*56*), we allow for some stochastic variation in the wording of the counterarguments of the LLM to make the output seem more natural. To provide an idea of how much the responses from the LLM vary, table S2 in text S3 provides three examples of responses to the same fictitious issue position for each treatment condition. The table shows that, while the wording and content vary slightly as expected, the overall substantive meaning of the three responses is very similar. In text S3, we also conduct validation checks for each of the four treatments to document that there is no overlap between conditions (e.g., evidence-based content in the emotion-based condition or vice versa) and that inaccurate information in counterargument due to “hallucinations” is rare.

### Outcome measures

After respondents were shown the response from the interlocutor, they were asked to respond to it as if writing a social media reply. To generate outcomes from these open-text replies, three coders manually coded them along dimensions analogous to the treatment dimensions themselves. Specifically, we code (i) whether a respondent’s reply is disrespectful (e.g., uses name-calling, pejoratives, and sarcasm); (ii) whether the reply provides a “qualified” justification (i.e., one with a clear link between a reason and a position) for their stance with reference to evidence or, alternatively, any justification, which includes “inferior” justifications (i.e., one lacking a clear link between a reason and a position) (*78*, *79*); (iii) whether the reply signals a willingness to compromise; and (iv) whether the reply includes a partisan attack [intercoder reliability scores (Krippendorff’s α) are as follows: justification = 0.83, disrespect = 0.82, compromise = 0.81, partisan attack = 0.81, and any partisan-related reference = 0.93]. Details on the preregistered coding scheme can be found in text S3.4.

Drawing on the existing literature on the elements of discourse that foster and hinder democratic discourse, as discussed in our theoretical framework above [e.g., (*26*, *36*, *39*–*42*, *46*, *47*, *52*, *80*)], we code replies from respondents as being of high quality if they fulfill the following criteria: (i) provide either a qualified justification or signal a willingness to compromise, (ii) are not disrespectful, and (iii) do not include a partisan attack. Otherwise, replies are coded as “low quality.” We also create an analogous measure for what we term a quality reply, where the standard for the justification criterion above is lowered to include replies that provide any justification (either qualified or inferior). In Results, we also examine the individual components of a high-quality (and quality) reply separately.

We then use a number of closed-form questions to test attitudinal and quasi-behavioral effects. First, we measure attitudes on the issue being discussed in terms of attitude strength and attitude certainty. To do so, we use the LLM to summarize each respondent’s (pretreatment) issue position and ask respondents the extent to which they agree or disagree with the summary and how certain they are about where they stand on that issue (*56*). To reduce ceiling effects, we prompt the LLM to summarize the respondent’s issue position in especially strong terms, using qualifiers such as “must,” “in all circumstances,” or “fully.” Following (*56*, *81*), we interpret decreases in attitude strength and certainty of the issue discussed as evidence of issue moderation.

Second, we measure political engagement by whether respondents are interested in learning more about arguments related to the issue at hand. To do this, we create a quasi-behavioral measure by asking respondents to click a link if they want further information about the issue. Using the LLM to summarize the issue topic, we ask whether a respondent “want[s] more information about arguments regarding [issue],” and present a hyperlink that reads “Click here if you would like more information about [issue].” To prevent contamination effects on the other outcomes and respondents dropping out of the survey, the link, which redirects to a Google search page about the topic, is provided only at the end of the survey.

Third, we measure openness to alternative viewpoints on the issue as an additive index of responses to three questions concerning whether there are valid reasons for having different views on the issue, whether issue opponents are less intelligent (reverse), and whether opponents on the issue do not know what they are talking about (reverse) (α = 0.76). Again, we use the LLM to summarize the issue position of the respondent. As noted in the preregistration, we excluded responses to the set of attitudinal and openness questions whether the LLM’s summary of the issue position was unclear or nonapplicable, as determined by a coding team (48 of 3301 cases), as well as responses regarding to the information link question whether the LLM’s summary of the issue was unclear or nonapplicable (54 of 3301 cases).

Fourth, we measure ideological polarization and affective polarization. Ideological polarization is measured as an additive index calculated as the average response to 10 questions about economic and cultural issues (α = 0.84). Higher values indicate more extreme positions ideologically (whether to the left or right) relative to an individual’s party identification [for a similar approach, see (*9*)]. Affective polarization is measured as the absolute difference between feelings toward the Republican and Democratic parties in the US and the Conservative and Labour parties in the UK [see (*55*) for example].

Last, we measure perceptions of a respondent’s interlocutor by asking respondents their impression of the fictitious user whose counterargument they received and replied to. These questions evaluated whether the interlocutor is disrespectful, is ideologically extreme, is well informed about politics, is open to changing their mind on the issue, makes reasonable arguments, makes strong arguments, and is engaging in constructive online political debate (for question wording of the survey questions, see text S3.1). In text S3.5, we provide descriptive statistics for all variables.

## Supplementary Materials

### This PDF file includes:

Texts S1 to S10 Tables S1 to S19 Figs. S1 to S30
