# Supplementary material for: Understanding the success and failure of online political debate: Experimental evidence using large language models
Source: Sci Adv. 2025 Jul 23;11(30):eadv7864. doi: 10.1126/sciadv.adv7864 (PMC12285710; doi:10.1126/sciadv.adv7864)
Supplement: Supplementary file 1 — Texts S1 to S10 Tables S1 to S19 Figs. S1 to S30 [file sciadv.adv7864_sm.pdf]

Science Advances
AAAS

Supplementary Materials for

Understanding the success and failure of online political debate: Experimental evidence using large language models

Tobias Heide-Jørgensen *et al.*

Corresponding author: Anne Rasmussen, anne.rasmussen@kcl.ac.uk

Sci. Adv. **11**, eadv7864 (2025)
DOI: 10.1126/sciadv.adv7864

This PDF file includes:

Texts S1 to S10
Tables S1 to S19
Figs. S1 to S30

Supplementary Text

S1. Ethics

Ethics approval was obtained by the Institutional Review Board at the University of Copenhagen (included in the replication data), and steps were taken to be ensure transparency with respondents regarding the purpose of the survey, its contents, and its use of a Large Language Model. Before participating, respondents were informed that the survey focused on dialogue on social media, and that they would be compensated monetarily from Prolific, the survey provider. Respondents were informed about the identities of the researchers involved; assured their anonymity would be respected in any research output; and told they could withdraw participation at any time. All respondents were asked to provide consent before starting the survey and were provided an email address to direct any questions about their participation. No deception was used in the survey. Respondents were told that they were interacting with a fictitious social media user and, during the debriefing, informed that this fictitious user was a Large Language Model. In the debriefing, respondents were also informed that they received a unique response from the Large Language Model depending on the issue that mattered to them and were told that the attributes of that response varied experimentally to allow researchers to understand how people react to different forms of political discourse on social media.

S2. Hypotheses

To facilitate communication in the theoretical and results sections, the order in which the hypotheses are introduced in the article is slightly different from the order in the pre-registration (https://osf.io/kc2u6/?view_only=c0b54cf90b5943c78405658b96514490.) Table S1 shows how the hypothesis numbers in the article relate to their numbering in the pre-registration. All hypotheses in the pre-registration are presented and tested in the article.

Table S1: **Difference in hypotheses numbering compared to the pre-registration**

Article hypothesis number	Pre-registration hypothesis number
1	1
2	2
3	5
4	4
5	6
6	3

Hypothesis 5 in the manuscript is substantively equivalent to that in the pre-registration, but for clarity is reversed in wording to talk about the treatments that are expected to reduce polarization, rather than increase it.

S3. Survey details and sample characteristics

S3.1. Question wording

In this section we present the wording of the main survey questions:

Issue position: "We now ask you to imagine that you want to write a post on social media (e.g., Facebook, Twitter, etc.) to express yourself on a political issue that you care about. Please write below, in a couple of sentences, your position on an issue that matters to you and briefly explain why. Your first sentence should state your position on the issue and the following sentences should explain why you have that position (please write in complete sentences). For example, if you care about small local businesses, you might write "The government should subsidize [in the UK: subsidise] small local businesses more. They are the backbone of our local communities."

Afterward, we will ask you to respond to a fictitious social media user who has responded to you and ask you some questions about it. Please note that it might take a while from when you click the 'next button' until the next page is shown."

Respondent reply: "If a social media user were to respond to your post like the fictitious user above, please type in below your response to them. Use at least a couple of sentences. Please read the post from the fictitious user carefully before you write your response."

Perceptions of social media user: "Based on your reading of the post from the fictitious user, what is your impression of that person? Please indicate how much you agree or disagree with the following statements." [strongly disagree, somewhat disagree, slightly disagree, slightly agree, somewhat agree, strongly agree]

- The user is disrespectful
- The user is ideologically extreme
- The user is well-informed about politics
- The user is open to changing their mind on the issue
- The user has reasonable arguments regarding the issue
- The user is engaging in constructive dialogue
- The user has strong arguments regarding the issue

Attitude strength, attitude certainty, openness to alternative beliefs: "Consider the following statement: [ChatGPT summary of respondent's issue position]" [strongly disagree, somewhat disagree, slightly disagree, slightly agree, somewhat agree, strongly agree]

Attitude strength regarding the issue discussed: "How much do you agree or disagree with the statement above?"

Attitude certainty regarding the issue discussed: "How certain are you about where you stand on this issue?

Openness items:

- "How much do you agree or disagree that there are valid reasons for having political views that differ from yours on this issue?"
- "How much do you agree or disagree that most people who think differently about this issue than you are not very intelligent?"
- "How much do you agree or disagree that people who disagree with you about this issue usually don't know what they are talking about?"

Information seeking: To measure information-seeking, we provided respondents with a hypertext link labeled "Click here if you would like more information about [issue topic]." When the link was clicked (which we recorded for measurement) we informed respondents that we had registered their interest in the topic and would redirect them to a site with further information at the survey's end. The link, provided at the end of the survey, took them to a Google search of the issue topic in major news media sites.

Affective polarization: "Please rate your feelings toward the Republican Party and the Democratic Party [in the UK: the Conservative Party and the Labour Party], with 0 meaning a very cold, unfavorable feeling, and 10 meaning a very warm, favorable feeling." [0 (Very cold), 1–9, 10 (Very warm)]

Ideological polarization: "How much do you agree or disagree with the following statements?" [strongly disagree, somewhat disagree, slightly disagree, slightly agree, somewhat agree, strongly agree]

- Differences in income levels are too large

- By law, a woman should always be able to obtain an abortion as a matter of personal choice
- Violent crimes should be punished much harsher than today
- The government should do more to financially support the poor
- Too many people these days like to rely on government handouts
- Homosexuality is always justifiable
- Hard-working people should be allowed to keep more of their earnings
- Illegal immigration is a big problem in the US today
- Economic growth and creating jobs should be the top priority, even if the environment suffers to some extent
- Government regulation of business is necessary to protect the public interest

S3.2. Large Language Model details

Expanding on the approach from (*56, 73*), the experiment manipulates various parts of a counter-argument tailored to each respondent's issue position and the reason behind that position. These counter-arguments were generated using independent calls to the ChatGPT-4 API to prevent crossover effects of the treatments.

These calls to the ChatGPT-4 API were done through programming within Qualtrics survey software, such that after respondents answer an open-text question about their position on an issue important to them (and their reasoning behind it) the ChatGPT-4 API was called to generate the treatment text. This text included sentences arguing the opposite position on the policy issue representing disrespect/respect, (non)partisan signaling (i.e., text framed as coming from an out-party, in-party, or non-partisan source), emotion-/evidence-based argumentation, and willingness or unwillingness- to compromise. We note that with respect to the in-party treatment, the LLM presents a co-partisan who may argue against the party line. This would reflect internal disagreement that common within political parties.

Examples of these treatment texts are highlighted separately in color in the main article in Figure 7. After Qualtrics received the responses from ChatGPT, the four treatment texts were pasted

together to form a single written vignette (as in Figure 7 in the main article). This setup is thus similar to a more standard full-factorial experimental vignette, but where the treatment texts are generated by an LLM and are therefore tailored to each respondent's open-text response. Although some order effects are possible, we did not randomize the order of the treatment texts because (1) it is difficult to seamlessly transition between LLM-generated sentences and (2) some of the ordering of treatment combinations is jarring and feel inorganic (e.g., starting by suggesting a compromise).

The prompts sent to the LLM to generate the treatment texts were tested using responses to a pilot survey in which respondents were asked for their position on an issue important to them and their reasoning behind it. The text of the prompts that generated text for each treatment condition were iterated on repeatedly until, qualitatively, the LLM's responses to the open-text answers that survey respondents provided in the pilot survey captured the treatment dimensions consistently. The prompts for each treatment condition were pre-registered. The prompt text for each treatment condition are made available in the pre-registration: `https://osf.io/kc2u6/?view_only=c0b54cf90b5943c78405658b96514490`.

LLM response consistency and validation

As noted above, the prompts for the LLM that generate the texts representing each treatment condition were developed through an iterative process using the issue positions as written by respondents in a pilot study. Substantial efforts were made to ensure that the prompts generated treatment texts that contained no overlap between conditions, e.g. that differences in the disrespect and respect conditions, emotion and evidence conditions, etc. are distinctly and consistently different. Each prompt, for instance, contains explicit instructions not to generate text related to another treatment condition (e.g. in the emotion-based argument condition, the prompt specifies the need to not provide any evidence and facts about the issue).

To validate this, we randomly selected 200 texts for each treatment condition (with equal numbers of texts from the UK and US samples) and created tasks for a research assistant to code differences in the LLM-generated texts. The research assistant coded each text for whether it indicated any element of its alternative: whether the compromise texts indicated any unwillingness to compromise; the uncompromising condition, any openness to compromise; the emotion-based condition any reference to evidence/reasoned argumentation; and for the evidence-based condition,

any reference to emotion-based argumentation. For the respect/disrespect condition, each text was coded on a 7-point scale to capture the extent to which it is disrespectful (i.e. where a 7 indicates "harsh sarcasm and ridicule" of a respondent's argument). For the partisanship condition, each text was classified by a research assistant for whether interlocutors explicitly identified themselves as a Labour supporter, Conservative, Democrat, or Republican, or did not indicate a partisan affiliation.

The results from this coding task show that outputs from the LLM are generated as expected. For the respect/disrespect conditions, all "respectful" texts were coded by a research assistant as a "1" on a 1-7 scale. In other words, all texts are essentially of the form "With respect, I firmly disagree with your stance on immigration." Among texts from the "disrespectful" condition, 81% were coded a "6" and 19% a "7", where a 7 indicates "harsh sarcasm and ridicule" of the respondent's argument. For the compromise/no compromise conditions, no elements of compromise were coded as present in the no compromise condition, and no elements of the unopen-to-compromise texts were coded as suggesting compromise. Similarly, for the evidence/emotion conditions, no elements of emotion were coded as present in the evidence-based texts, and no evidence or reasoned argumentation were coded as present in the emotion-based texts. Finally, with respect to partisanship, all texts assigned to come from Labour, Conservative, Democrat, or Republican supporters were coded as indicating the correct party, or with indicating no party (as in the non-partisan condition).

To show qualitatively how the output of the LLM might vary when using the treatment condition prompts and the same issue position, we present in Table S2 three responses to each LLM treatment condition prompt based on equivalent calls to the ChatGPT-4 API. For the example shown in Table S2, all responses are those to the following issue position: "The government needs to crack down on immigration by preventing all illegal immigration and only allowing entry to those who apply legally." Note that we display only one partisan category (rather than separate in-party and out-party categories, as in the paper) as the content is identical except for references to "Republican" or "Democrat" (in the US case). As Table S2 demonstrates, while the wording and sentence structure differ slightly across the three examples, the content and style are largely consistent. As noted in the article, one can think of this as analogous to stimulus sampling, where each text is a sample from a range of possibilities that capture the treatment of interest. This helps illustrate how the model tends to generate responses that are similar in meaning and tone, even if the exact content and sentence structure vary, albeit relatively minimally. Since LLMs generate texts proba-

bilistically, some variation is expected. However, the overall consistency across responses suggests that they can reasonably be treated as equivalent examples of the same treatment condition.

Finally, we investigate the extent to which the texts generated for the evidence-based treatment condition resulted in any "hallucinations" by the LLM, i.e. reference to, for example, studies, arguments, or facts that lack any actual evidentiary foundation. To examine this, we randomly selected 100 of the evidence-based texts in the data as generated by the LLM, and created a validation task in which a research assistant was instructed to spend (up to) 10 minutes per text to document and validate each evidence-based argument. The arguments were classified and documented as having evidence consistent with the LLM's argument, having no evidence found in support of the LLM's argument, or containing information that is contradicted by the factual record. This task resulted in one instance of a claim that is likely inaccurate: that a study shows that a majority of doctors no longer accept Medicaid patients due to low reimbursement rates. Specifically, although it is true that doctors accept many fewer patients on Medicaid than on Medicare or with private insurance, no study could be found stating that it is a *majority* of doctors (and government documents show that it is less than a majority). Although this task found that "hallucinations" in the counter-arguments generated by the LLM are rare, it should be kept in mind that the inaccuracy of evidence-based claims may affect responses.

Other uses of the LLM

For a small number of closed-form survey questions, we use ChatGPT-4 to summarize the issue/topic most important to each respondent, and to summarize the position they hold.

These summaries are for the survey questions in which the survey first shows "Consider the following statement: [ChatGPT summary of respondent's issue position]", which is followed by questions concerning attitude strength and regarding the issue, and openness to alternative perspectives. As noted in the main article, the prompt used to generate this summary specifies that the issue position should be stated in exceptionally strong terms, to avoid ceiling effects. The specific wording of the prompt for the LLM was the following:

> In simple language, summarize in a statement the following person's position on an issue in a single short sentence (maximum 30 words). The summary of the issue position should be a single sentence that someone in a survey could be asked to agree or disagree with. The person's stated position is the

Table S2: **Variation in ChatGPT responses to the same issue position.** Three examples of responses to fictitious issue position: "The government needs to crack down on immigration by preventing all illegal immigration and only allowing entry to those who apply legally.", using the relevant US prompts for each treatment condition.

**Treatment**	**Response 1**	**Response 2**	**Response 3**
Respectful	With respect, I firmly disagree with your stance on immigration.	Respectfully, I strongly disagree with your stance on immigration.	Respectfully, I strongly disagree with your views on immigration.
Disrespectful	Wow, you've solved it all with that simple solution! Genius! - Absurd.	Wow, such an original idea! Let's also stop all crime by just making everything illegal. Absurd.	Wow, what a groundbreaking idea! Let's just solve all problems by oversimplifying them. Absurd!
Partisan	As a Republican, I think you're wrong on this.	As a Republican, I think you're wrong on this.	As a Republican, I think you're wrong on this.
Non-partisan	I think you're wrong about this.	I think you're wrong about this.	I think you're wrong on this.
Evidence-based	Studies show that immigrants, both legal and illegal, contribute positively to the economy through labor force growth, entrepreneurship, and paying taxes. Blocking immigration could harm the economy.	Studies show that immigrants, including those undocumented, contribute significantly to the economy through taxes and consumption, and fill labor gaps in key industries.	Research shows that immigrants, both legal and illegal, contribute significantly to the economy and are less likely to commit crimes than native-born citizens. Your solution oversimplifies a complex issue.
Emotional	It makes me incredibly upset that we can't unanimously reject the idea of an absolute crackdown on immigration. It's frustrating that not everyone understands why this is wrong for our economy.	It makes me incredibly upset that some believe that an all-out crackdown on immigration is the answer. It's frustrating that not everyone realizes why this perspective is harmful for our economy.	It makes me incredibly upset that such an extreme stance on immigration is considered a solution. It's disheartening to see the lack of understanding about its broader implications on our economy.
Compromise	We see this issue differently, but I'm willing to chat and find some middle ground.	We clearly don't see eye to eye on this, but I'm open to finding some common ground.	We clearly see this differently, but maybe we can still discuss to find some common ground.
No compromise	Clearly, we're at odds here and I don't see us finding a middle ground on this issue.	We clearly have big differences on this issue, so trying to reach a middle ground seems not worth the effort.	Clearly, we don't see eye to eye on this matter, so it's not worth trying to reach an agreement.

following: [RESPONDENT'S ARGUMENT] Please also do the following: Do not use quotation marks around the response. Capitalize the first letter of the first word and proper nouns but otherwise use only lowercase letters. Do not ask in the statement whether someone agrees or disagrees with it. Do not indicate the reason why someone should agree or disagree with the statement. Do not refer to the person who stated the issue position. Do not refer to the person stating the issue position even if their

> position includes the word “I” or “my”. Do not state whether the person agrees or disagrees with the issue position. Do not state how the person or people in general see the issue. Do not use the same emotional or strong language about the issue as the person. Make the statement so that it is only one side of the issue. Do not refer to the person’s opinion about the issue. Make the issue position one that one might need to be extreme to agree or disagree with fully. State the issue position in the strongest possible terms using very strong qualifiers like “must” and/or “in all circumstances” and/or “fully” and/or “ban” and/or “unrestricted” and/or “absolutely” and/or “totally” and/or “it is never” and/or “it is always” and/or “far more” and/or “far less” and/or “completely” and/or “strict” and/or “no matter the cost” and/or “regardless of the cost”. Do not refer to things like “better” policy or “highest quality” that almost everyone would agree with. Where possible, use declarative language like “will” rather than softer language like “could”, “potentially”, or “might”. Use simple language.

Finally, the survey questions include one in which we ask whether respondents would like to learn more about arguments regarding the issue important to them, providing a hyperlink for them to click if they are interested. The text of the link reads “Click here if you would like more information about [issue topic].” To generate the text of the issue topic, we used the following prompt for the LLM:

> Use 1-4 words to summarize what political issue the following argument is about: [RESPONDENT’S ARGUMENT] Don’t mention a specific position, just the overall issue. Do not use quotation marks around the response. Do not use full stop, comma, question mark, exclamation mark, etc. Use only lowercase letters, unless it is a proper noun.

S3.3. Policy issues

Based on ChatGPT’s brief summary of the issue area of respondents’ initial text, we manually grouped these into more aggregate issues to get an overview of the topics that respondents found important and wanted to express an opinion about. To give an example, public education, private education, kindergarten, etc. were combined into one overall education category. Overall, 112 unique issue areas were identified. Table S3 shows the ten most frequently occurring issues. We see that abortion, healthcare, and immigration are the three most popular issues, but also that the most frequent issue (abortion) only accounts for roughly 15% of all cases.

S3.4. Coding replies

Three coders hand-coded all open-text replies from respondents using a pre-registered codebook, which is attached to the pre-registration at: (Files → Archive of OSF Storage → Codebook, `https:`

Table S3: **Top 10 issue topics (%).** The 10 most frequent topics among respondents. Aggregated issues based on ChatGPT's summary of the issue area of respondents' post. Cases where ChatGPT's issue summary was manually coded as invalid (n = 54) are excluded.

Category	Percent
Abortion	14.5%
Healthcare and public health	8.2%
Immigration	7.9%
Climate change	7.0%
Education	5.5%
Gun control	4.7%
Taxes	3.7%
Gender issues	3.5%
Environment	3.1%
Cost of living and inflation	2.9%
N	3251

`//osf.io/kc2u6/?view_only=c0b54cf90b5943c78405658b96514490`). Replies were coded for whether they (1) included a "qualified" justification (2) were disrespectful, (3) signaled a willingness to compromise, and (4) included a partisan attack. As secondary measures, replies were coded for whether they provided an "inferior" justification and included any mentions of a political party or politician. In cases of disagreement, a decision was made by a majority of the coders. In the rare cases in which the three coders were split between whether a reply included no justification, an "inferior" justification, or a "qualified" justification, the reply was coded as giving an "inferior" justification. Inter-coder reliability for each of the coded outcomes is the following: justification (ordinal, $\alpha = 0.83$); disrespect ($\alpha = 0.82$); compromise ($\alpha = 0.81$); partisan attack ($\alpha = 0.81$); partisanship-related mentions ($\alpha = 0.93$).

S3.5. Descriptive statistics

Table S4 presents descriptive statistics for all variables used in the main article.

S3.6. Representativeness

In this section, we assess the representatives of our US and UK samples, benchmarking them against

Table S4: **Descriptive statistics.** SD: standard deviation, Min: minimum value, Max: maximum value.

	Mean	SD	Min	Max	Observations
Tone					
Respectful tone	0.49	0.50	0.00	1.00	3,305
Disrespectful tone	0.51	0.50	0.00	1.00	3,305
Justification					
Evidence-based argument	0.50	0.50	0.00	1.00	3,305
Emotional argument	0.50	0.50	0.00	1.00	3,305
Compromise					
Compromise	0.51	0.50	0.00	1.00	3,305
No compromise	0.49	0.50	0.00	1.00	3,305
Partisanship					
In-party	0.33	0.47	0.00	1.00	3,305
Out-party	0.34	0.47	0.00	1.00	3,305
Non-partisan	0.34	0.47	0.00	1.00	3,305
High quality reply (binary)	0.35	0.48	0.00	1.00	3,303
Quality reply (binary)	0.65	0.48	0.00	1.00	3,303
High quality reply (continuous)	2.28	0.74	0.00	4.00	3,303
Quality reply (continuous)	2.69	0.79	0.00	4.00	3,303
Disrespectful (reply)	0.08	0.28	0.00	1.00	3,303
Qualified justification (reply)	0.15	0.36	0.00	1.00	3,303
Any justification (reply)	0.56	0.50	0.00	1.00	3,303
Compromise (reply)	0.24	0.43	0.00	1.00	3,303
Partisan attack (reply)	0.04	0.19	0.00	1.00	3,303
Any mentions of party (reply)	0.11	0.31	0.00	1.00	3,303
Seek info	0.50	0.50	0.00	1.00	3,251
Attitude strength	0.94	0.16	0.00	1.00	3,230
Attitude certainty	0.94	0.13	0.00	1.00	3,231
Openness index	0.54	0.26	0.00	1.00	3,231
Affective polarization	0.00	1.00	-1.78	1.89	3,275
Ideological polarization	0.00	1.00	-4.05	2.33	3,271
Disrespectful perception	0.49	0.35	0.00	1.00	3,278
Extreme perception	0.56	0.31	0.00	1.00	3,278
Informed perception	0.36	0.28	0.00	1.00	3,280
Compromise perception	0.26	0.30	0.00	1.00	3,281
Constructive perception	0.41	0.33	0.00	1.00	3,280
Reasonable arguments perception	0.33	0.30	0.00	1.00	3,280
Strong arguments perception	0.39	0.34	0.00	1.00	3,280

American and British election samples. Table S5 compares the US sample with respondents from the 2020 American National Election Study (ANES), and Table S6 compares the UK sample with respondents from the 2019 British Election Study (BES). Overall, our samples are younger and

include a higher proportion of more male respondents compared to the election study samples. In addition, our US sample is slightly worse educated than the ANES sample, and our UK sample is slightly better educated than the BES sample.

Table S5: **Comparing the US sample with ANES respondents (%).** "ANES" is the 2020 American National Election Study. Only respondents in ANES that feel close to the Democratic Party or the Republican Party are used to match the sample criteria used for our study.

	**US Sample**	**ANES**
Gender		
Male	50.37	45.89
Female	49.63	54.11
Age		
18-35	42.44	19.84
36-59	44.65	39.64
60+	12.92	40.52
Education		
Primary school / no formal education	1.42	3.23
High school / some college	30.41	34.57
Vocational training / community college	13.02	7.20
Undergraduate education	39.58	32.98
Postgraduate education	15.57	22.01
Observations	1,766	5,810

S3.7. Treatment group balance tables

To check for balance in observed characteristics across treatment conditions, we present in Tables S7, S8, S9, and S10, the differences in sample characteristics for respondents assigned to each of the 4 treatments. We also present p-values for comparisons between the treatment conditions and each respondent characteristic. As the tables show, there are no meaningful differences in treatment assignment across these observed characteristics (among all tables, only one difference is statistically significant).

S3.8. Differences in attrition by treatment condition

It may be the case that survey respondents were more unwilling to respond to certain replies gen-

Table S6: **Comparing UK sample and BES respondents (%).** "BES" is the 2019 British Election Study. Only respondents in BES that feel close to the Labour Party or the Conservative Party are used to match the sample criteria used for our study.

	**UK Sample**	**BES**
Gender		
Male	49.80	47.99
Female	50.20	52.01
Age		
18-35	37.36	20.05
36-59	45.55	39.31
60+	17.09	40.65
Education		
Primary school / no formal education	0.26	20.88
GCSE or below	15.72	11.56
A-level	25.86	17.72
Undergraduate education	40.87	37.52
Postgraduate education	17.28	12.33
Observations	1,539	1,347

erated by the LLM depending on the treatment conditions. For example, respondents may be less likely to write responses to counter-arguments that are disrespectful, resulting in attrition bias. To test this, we estimate the effect of each treatment condition on whether respondents wrote an open-text reply to the counter-argument as requested in the survey. Results are presented in Table S11. We find no evidence that respondents were more or less likely to write replies to counter-arguments generated through the different treatments.

Table S7: **Balance table comparing differences in assignment to the disrespectful/respectful treatment conditions**

	Respectful (N=1618)		Disrespectful (N=1687)			
	**Mean**	**Std. Dev.**	**Mean**	**Std. Dev.**	**Diff. in Means**	**p**
Woman	0.50	0.50	0.50	0.50	-0.00	0.85
Aged 18-35	0.40	0.49	0.40	0.49	-0.00	0.91
Aged 36-59	0.46	0.50	0.45	0.50	-0.01	0.58
Aged 60+	0.14	0.35	0.15	0.36	0.01	0.36
High school / some college	0.22	0.42	0.25	0.43	0.02	0.09
Vocational training / community college	0.19	0.39	0.19	0.39	0.00	0.97
Undergraduate education	0.41	0.49	0.39	0.49	-0.02	0.36
Postgraduate education	0.17	0.37	0.16	0.37	-0.01	0.63
Party ID: Conservative	0.23	0.42	0.22	0.41	-0.01	0.66
Party ID: Labour	0.25	0.43	0.24	0.43	-0.01	0.72
Party ID: Democrat	0.27	0.44	0.26	0.44	-0.01	0.71
Party ID: Republican	0.26	0.44	0.28	0.45	0.02	0.26
Ideological self-identification (1-7 scale)	3.28	1.51	3.32	1.51	0.03	0.51
Social media use (30+ minutes a day)	0.54	0.50	0.54	0.50	-0.00	0.80

* $p < 0.05$, ** $p < 0.01$, *** $p < 0.001$.

Table S8: **Balance table comparing differences in assignment to the evidence/emotion treatment conditions**

	Emotional argument (N=1645)		Scientific argument (N=1660)			
	**Mean**	**Std. Dev.**	**Mean**	**Std. Dev.**	**Diff. in Means**	**p**
Woman	0.50	0.50	0.50	0.50	-0.00	0.93
Aged 18-35	0.41	0.49	0.39	0.49	-0.01	0.40
Aged 36-59	0.44	0.50	0.46	0.50	0.03	0.14
Aged 60+	0.15	0.36	0.14	0.35	-0.01	0.35
High school / some college	0.23	0.42	0.24	0.43	0.01	0.47
Vocational training / community college	0.19	0.39	0.19	0.39	-0.01	0.69
Undergraduate education	0.40	0.49	0.40	0.49	-0.01	0.72
Postgraduate education	0.16	0.37	0.16	0.37	0.00	0.90
Party ID: Conservative	0.22	0.41	0.23	0.42	0.01	0.63
Party ID: Labour	0.24	0.43	0.24	0.43	0.00	0.92
Party ID: Democrat	0.27	0.44	0.27	0.44	-0.00	0.84
Party ID: Republican	0.27	0.44	0.26	0.44	-0.01	0.72
Ideological self-identification (1-7 scale)	3.29	1.51	3.31	1.51	0.02	0.65
Social media use (30+ minutes a day)	0.54	0.50	0.54	0.50	-0.01	0.67

* $p < 0.05$, ** $p < 0.01$, *** $p < 0.001$.

Table S9: **Balance table comparing differences in assignment to the compromise treatment conditions**

	No compromise (N=1620)		Compromise (N=1685)			
	**Mean**	**Std. Dev.**	**Mean**	**Std. Dev.**	**Diff. in Means**	**p**
Woman	0.48	0.50	0.51	0.50	0.03	0.07
Aged 18-35	0.40	0.49	0.40	0.49	0.01	0.73
Aged 36-59	0.45	0.50	0.45	0.50	-0.00	0.92
Aged 60+	0.15	0.36	0.15	0.35	-0.00	0.74
High school / some college	0.24	0.43	0.23	0.42	-0.01	0.56
Vocational training / community college	0.19	0.39	0.19	0.39	-0.00	0.85
Undergraduate education	0.39	0.49	0.41	0.49	0.01	0.40
Postgraduate education	0.17	0.37	0.16	0.37	-0.00	0.72
Party ID: Conservative	0.23	0.42	0.22	0.41	-0.00	0.75
Party ID: Labour	0.25	0.43	0.24	0.43	-0.00	0.75
Party ID: Democrat	0.27	0.44	0.27	0.44	-0.00	0.80
Party ID: Republican	0.26	0.44	0.27	0.45	0.01	0.39
Ideological self-identification (1-7 scale)	3.29	1.51	3.31	1.52	0.02	0.71
Social media use (30+ minutes a day)	0.55	0.50	0.54	0.50	-0.01	0.65

* p < 0.05, ** p < 0.01, *** p < 0.001.

Table S10: **Balance table comparing differences in assignment to the partisanship treatment conditions** Because there are more than 2 treatment conditions for the partisanship treatment, p-values are calculated from χ^2 and ANOVA tests for categorical and continuous characteristics respectively.

	In-party (N=1077)		Out-party (N=1110)		Non-partisan (N=1118)		
	**Mean**	**Std. Dev.**	**Mean**	**Std. Dev.**	**Mean**	**Std. Dev.**	**p**
Woman	0.47	0.50	0.51	0.50	0.51	0.50	0.11
Aged 18-35	0.43	0.50	0.39	0.49	0.38	0.49	0.04 *
Aged 36-59	0.43	0.49	0.46	0.50	0.47	0.50	0.15
Aged 60+	0.14	0.35	0.15	0.36	0.15	0.36	0.70
High school / some college	0.24	0.43	0.24	0.43	0.23	0.42	0.61
Vocational training / community college	0.20	0.40	0.18	0.38	0.20	0.40	0.37
Undergraduate education	0.39	0.49	0.41	0.49	0.41	0.49	0.39
Postgraduate education	0.17	0.37	0.16	0.37	0.16	0.37	0.84
Party ID: Conservative	0.24	0.43	0.22	0.41	0.21	0.41	0.14
Party ID: Labour	0.22	0.42	0.25	0.43	0.25	0.43	0.21
Party ID: Democrat	0.26	0.44	0.27	0.45	0.27	0.44	0.81
Party ID: Republican	0.27	0.45	0.25	0.44	0.27	0.45	0.56
Ideological self-identification (1-7 scale)	3.34	1.51	3.29	1.52	3.26	1.51	0.45
Social media use (30+ minutes a day)	0.52	0.50	0.54	0.50	0.56	0.50	0.14

* p < 0.05, ** p < 0.01, *** p < 0.001.

Table S11: **Difference in attrition by treatment condition.**

	Whether respondent replied to counter-argument
Evidence-based argument (ref.: emotional)	0.002
	(0.003)
Compromise (ref.: no compromise)	0.006
	(0.003)
Disrespectful tone (ref.: respectful)	0.001
	(0.003)
Out-party (ref.: in-party)	-0.003
	(0.004)
Non-partisan (ref.: in-party)	-0.002
	(0.004)
(Intercept)	0.007
	(0.004)
N	3305

* p < 0.05, ** p < 0.01, *** p < 0.001

S4. Mobilization

Figure S1 shows the effects of the treatments on information search in the form of clicking a link to further information about the issue, with the complete regression table provided in Table S12. No treatment condition has a significant effect on whether a respondent seeks out more information about the issue at hand.

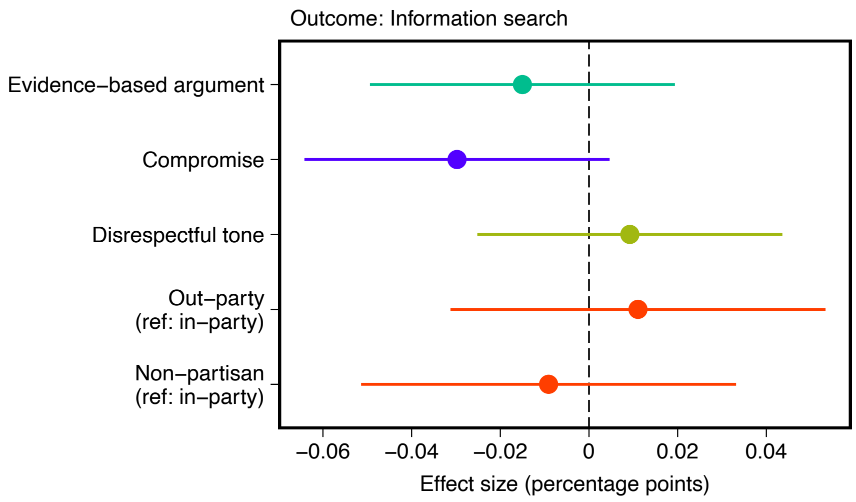

Figure S1: **Effects of attributes of counter-argument on probability of clicking information link.** Estimated treatment effects with 95% confidence intervals from OLS regression model that includes all treatment variables.

Table S12: **Effects of attributes of counter-argument on probability of clicking information link.** Regression estimates with standard errors in parentheses.

	Information search
Evidence-based argument (ref.: emotional)	-0.015
	(0.018)
Compromise (ref.: no compromise)	-0.030
	(0.018)
Disrespectful tone (ref.: respectful)	0.009
	(0.018)
Out-party (ref.: in-party)	0.011
	(0.022)
Non-partisan (ref.: in-party)	-0.009
	(0.022)
(Intercept)	0.517***
	(0.022)
Observations	3251

* p < 0.05, ** p < 0.01, *** p < 0.001

S5. How perceptions predict the quality of a reply

Figure S2 shows how perceptions of the interlocutor predict the probability that the respondent gives a high-quality response. Perceiving the interlocutor as giving reasonable arguments and being open-minded increases the likelihood of a high-quality response, whereas viewing the user as disrespectful decreases the likelihood of a high-quality reply. Combined with Figure 6 in the main article, which shows that the treatments affect perceptions of the interlocutor, this suggests that perceptions of the discussion partner are a plausible mechanism behind the effect of discourse on debate quality.

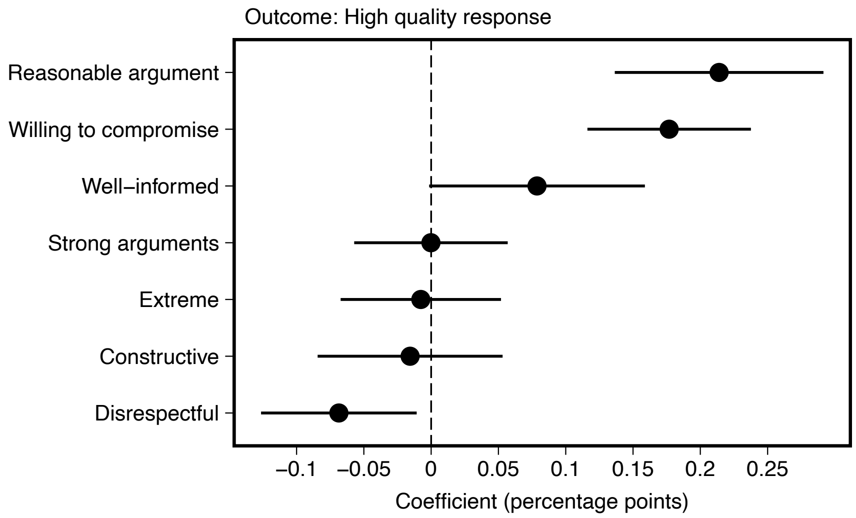

Figure S2: **Association between perceptions of interlocutor and probability of high-quality reply.** Predicted association with 95% confidence intervals from OLS regression model that includes all perception variables.

S6. Results by country

In this section, we present results for the primary results in the main article, broken down by whether a respondent is from the United States or United Kingdom. As the figures show, results are very similar between countries, although we note any significant differences in each of the subsections below.

S6.1. Quality of reply

Figure S3 and Figure S4 present results for the effect of each treatment on whether the respondent provides a "High quality" (binary) or "Quality" (binary) response. These figures are analogous to Figure 1 in the main article. Testing for differential effects between the treatments and a respondents' country shows no significant differences.

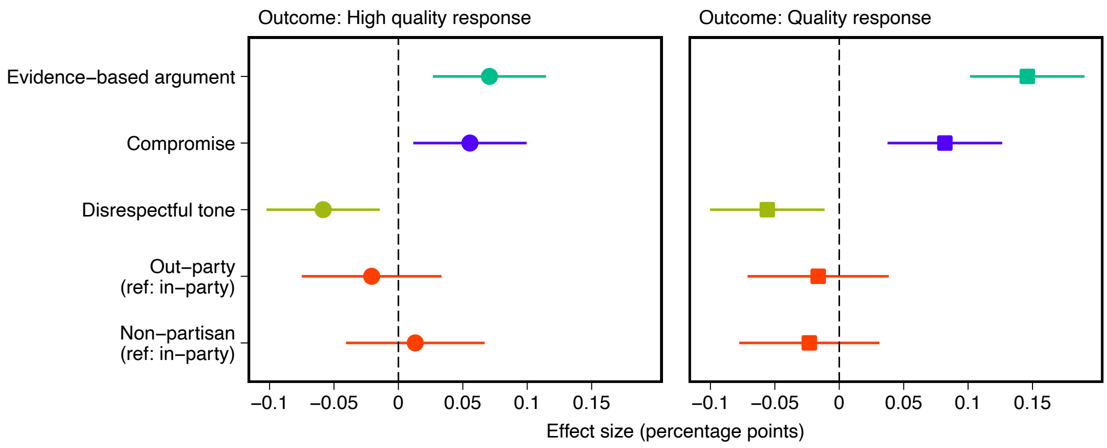

Figure S3: **United States: Effects of attributes of counter-argument on probability of high-quality reply.** Estimated treatment effects with 95% confidence intervals from OLS regression models that include all treatment variables.

S6.2. Detailed discourse-level outcomes

Figure S5 and Figure S6 present results for the effect of each treatment on the elements of a respondents' response: whether their response provides a "High quality" or "Any" argument; whether it indicates willingness to compromise; and whether it contains a partisan attack. These figures are analogous to Figure 2 in the main article. Testing for differential effects of the treatments by a

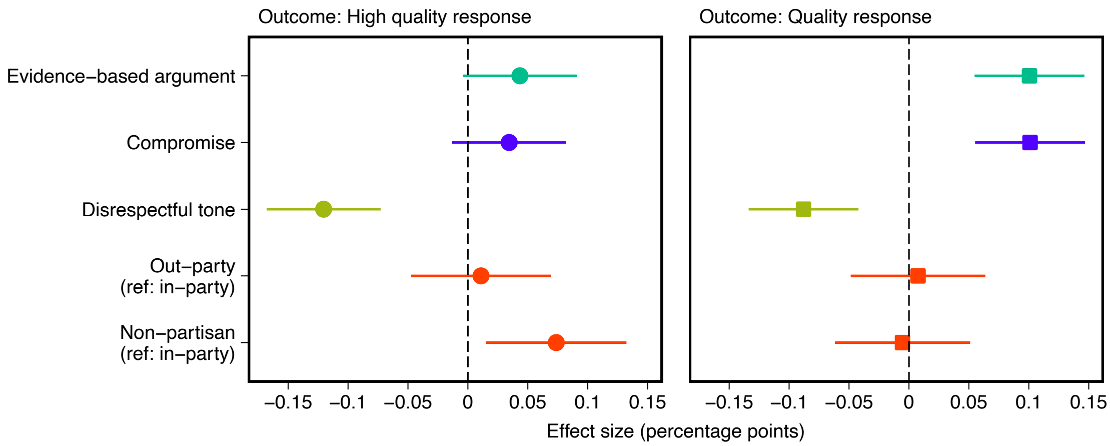

Figure S4: **United Kingdom: Effects of attributes of counter-argument on probability of high-quality reply.** Estimated treatment effects with 95% confidence intervals from OLS regression models that include all treatment variables.

respondent's country shows one significant difference: the negative effect of the disrespect treatment on a respondent indicating their willingness to compromise is larger in the UK than in the US (p = 0.02). We note, however, that this is one significant difference out of twenty-five tests (nominally less than we would expect by chance under the null hypothesis of no country-specific differences).

S6.3. Attitude moderation

Figure S7 and Figure S8 present results for the effect of each treatment on changes in the strength of respondents' position on the issue they believe is most important and their certainty about that position. These figures are analogous to the results discussed in the "Political Orientations" subsection of the Results in the main article. Testing for differential effects of the treatments by a respondent's country shows two significant differences: the effect of the disrespectful treatment negatively affects the strength of a respondent's attitude towards the issue in the UK compared to the US (p = 0.04); and the non-partisan treatment is significantly larger in the US compared to the UK (p = 0.04).

S6.4. Openness to other perspectives

Figure S9 and Figure S10 present the treatment effects on whether respondents are more or less

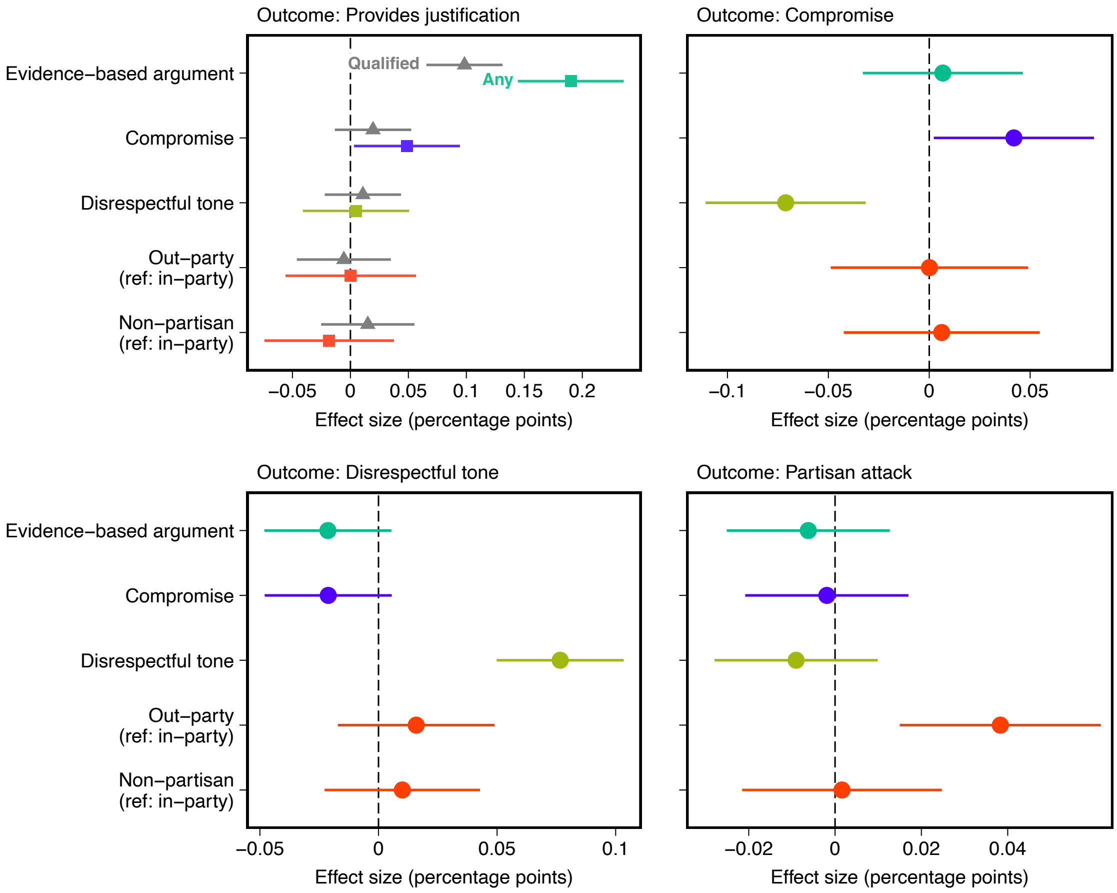

Figure S5: **United States: Effects of attributes of counter-argument on content and tone of reply.** Estimated treatment effects with 95% confidence intervals from OLS regression models that include all treatment variables.

open to alternatives perspectives on the issue. These figures are analogous to Figure 3 in the main article. We observe no significantly different effects for any treatment by country.

S6.5. Polarization

Figure S11 and Figure S12 present results for the effect of each treatment on whether respondents are more or less polarized on their ideological issue positions, or more or less affectively polarized. These figures are analogous to results presented in Figure 4 in the main article. Testing for differential treatment effects shows no significant differences for any treatments by country.

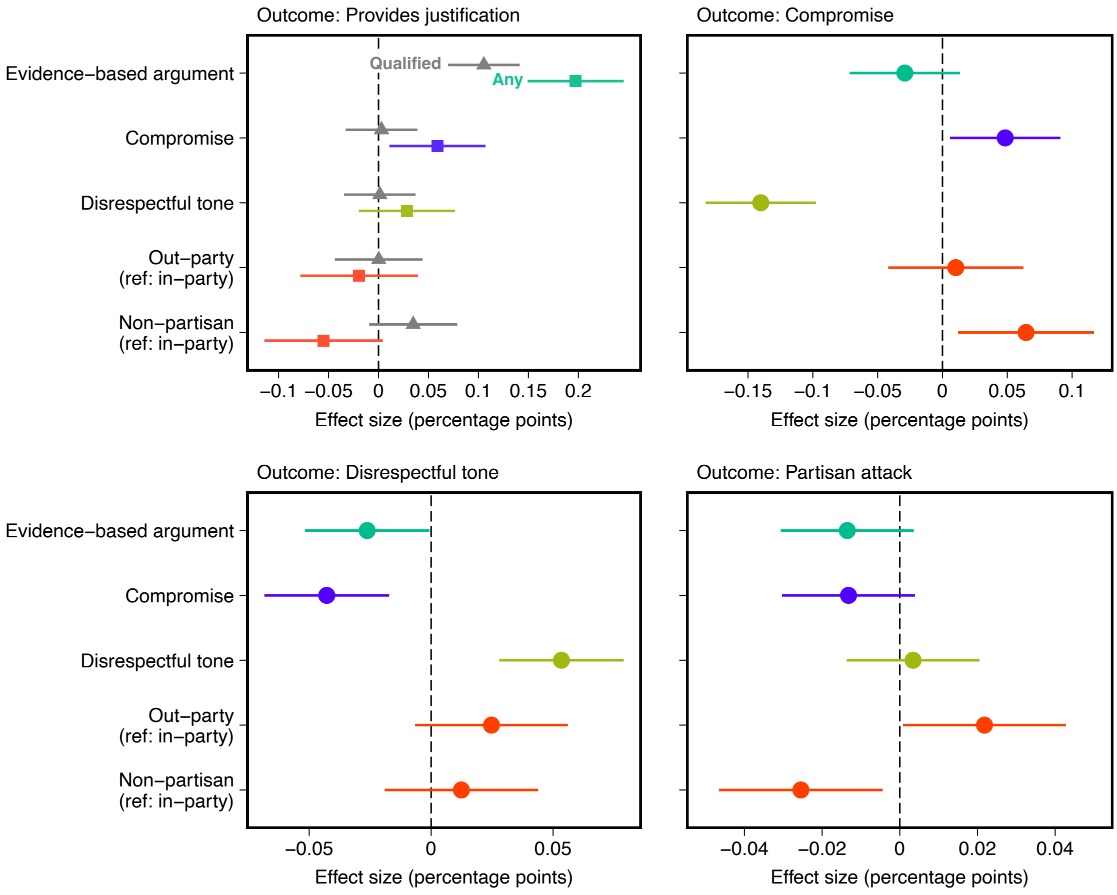

Figure S6: **United Kingdom: Effects of attributes of counter-argument on content and tone of reply.** Estimated treatment effects with 95% confidence intervals from OLS regression models that include all treatment variables.

S6.6. Information search

Figure S13 and Figure S14 present results for the effect of each treatment on whether respondents seek out information on their chosen political issue of interest. These figures are analogous to results presented in the "Political engagement" subsection of the Results section in the main article. Testing for differential effects shows no significant differences for any treatments by country.

S6.7. Perceptions of the interlocutor

Figure S15 and Figure S16 present the effects of the treatments on respondents' perceptions of the (fictitious) person who responded to them with a counter-argument. These figures are analogous to

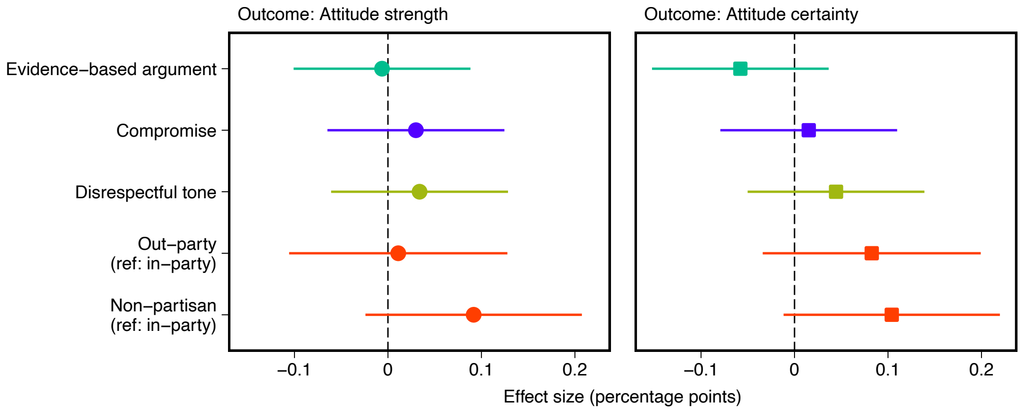

Figure S7: **United States: Effects of attributes of counter-argument on attitude strength and attitude certainty regarding the issue.** Estimated treatment effects with 95% confidence intervals from OLS regression models that include all treatment variables.

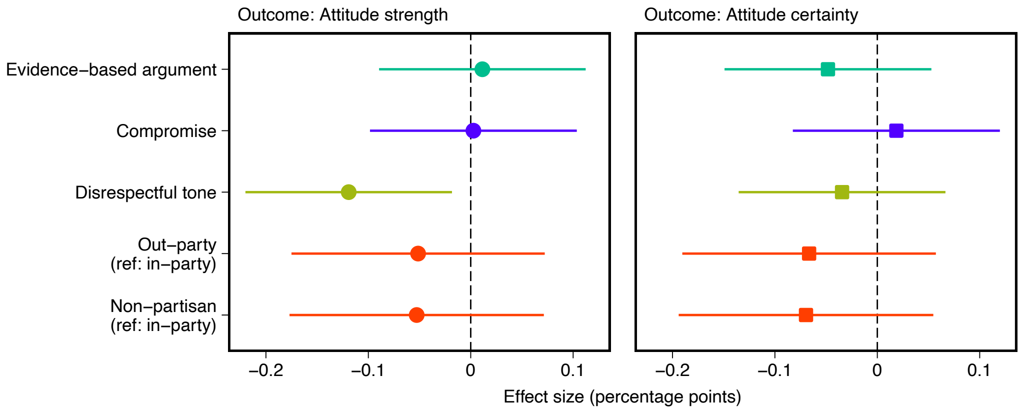

Figure S8: **United Kingdom: Effects of attributes of counter-argument on attitude strength and attitude certainty regarding the issue.** Estimated treatment effects with 95% confidence intervals from OLS regression models that include all treatment variables.

Figure 6 in the main article. Testing for differential effects of the treatments on respondents' perceptions of their interlocutor by country reveals several significant differences. In assessments whether an interlocutor makes good arguments or is being constructive, there are no significant differences between the US and UK. In assessments of whether an interlocutor is open-minded, the compromise treatment has a larger positive effect in the UK than the US (p = 0.02). In assessments of whether an interlocutor is well-informed, the scientific argument treatment has a larger positive effect in the

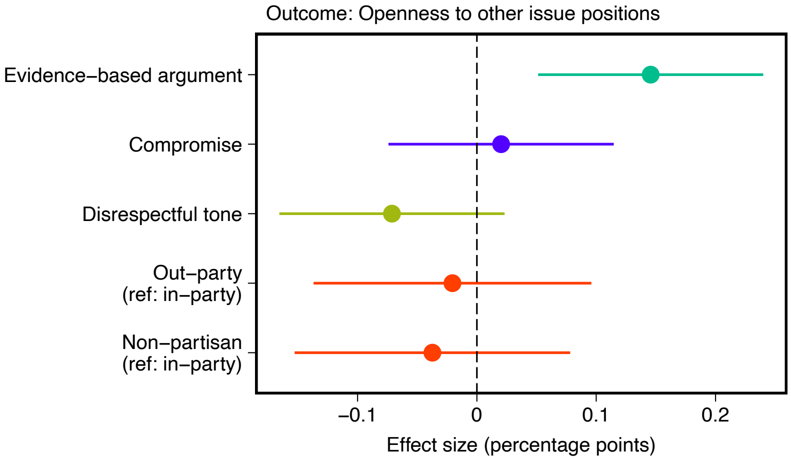

Figure S9: **United States: Effects of attributes of counter-argument on openness to other beliefs.** Estimated treatment effects with 95% confidence intervals from OLS regression model that includes all treatment variables.

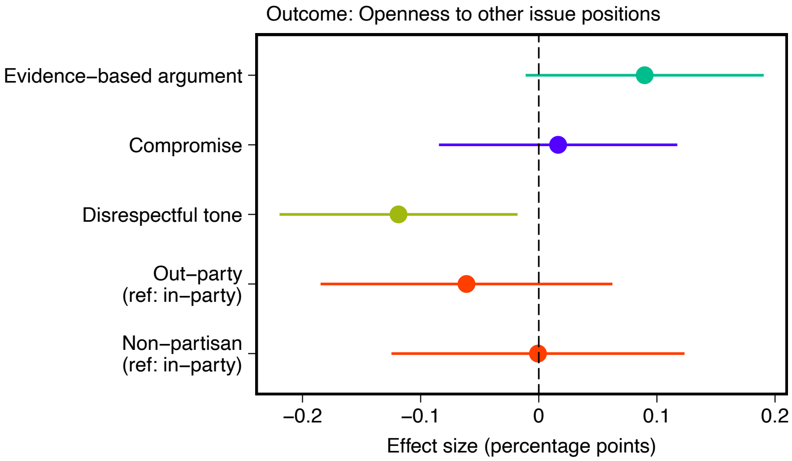

Figure S10: **United Kingdom: Effects of attributes of counter-argument on openness to other beliefs.** Estimated treatment effects with 95% confidence intervals from OLS regression model that includes all treatment variables.

UK than the US ($p = 0.03$). In contrast, in assessments of whether an interlocutor is disrespectful, the disrespectful treatment has a larger positive effect in the US than the UK ($p < 0.001$). Finally, in assessments of whether the interlocutor is ideologically extreme, the out-party and non-partisan treatments have larger positive effects in the US than in the UK ($p = 0.02$, $p = 0.04$ respectively). We stress, however, that these comparisons should be treated as exploratory given the large number

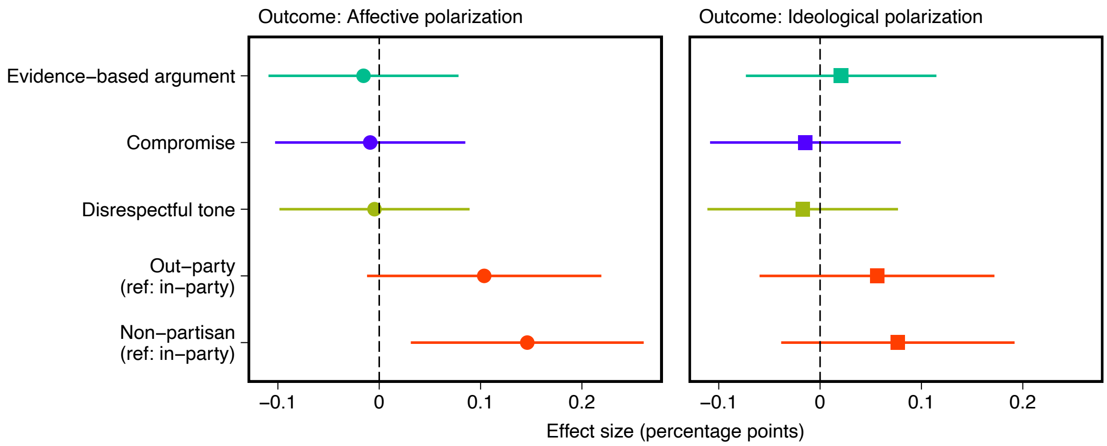

Figure S11: **United States: Effects of attributes of counter-argument on affective and ideological polarization.** Estimated treatment effects with 95% confidence intervals from OLS regression models that include all treatment variables.

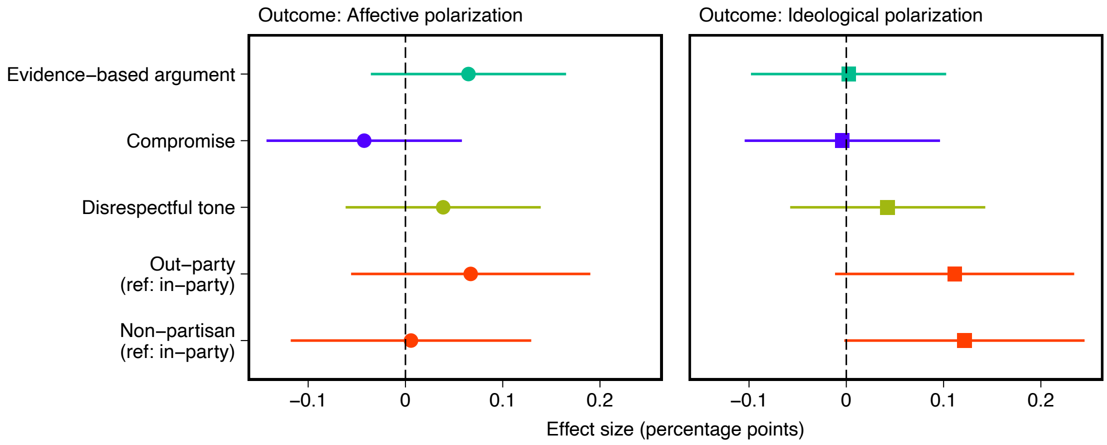

Figure S12: **United Kingdom: Effects of attributes of counter-argument on affective and ideological polarization.** Estimated treatment effects with 95% confidence intervals from OLS regression models that include all treatment variables.

of interaction terms tested.

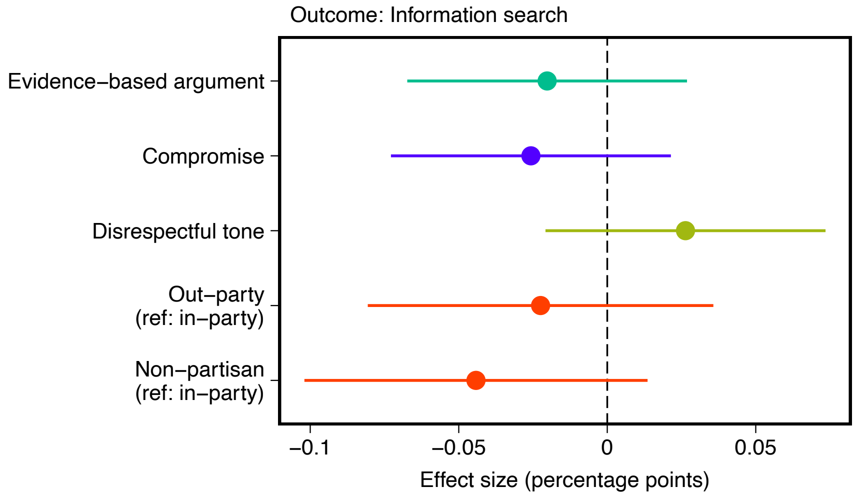

Figure S13: **United States: Effects of attributes of counter-argument on probability of clicking information link.** Estimated treatment effects with 95% confidence intervals from OLS regression model that includes all treatment variables.

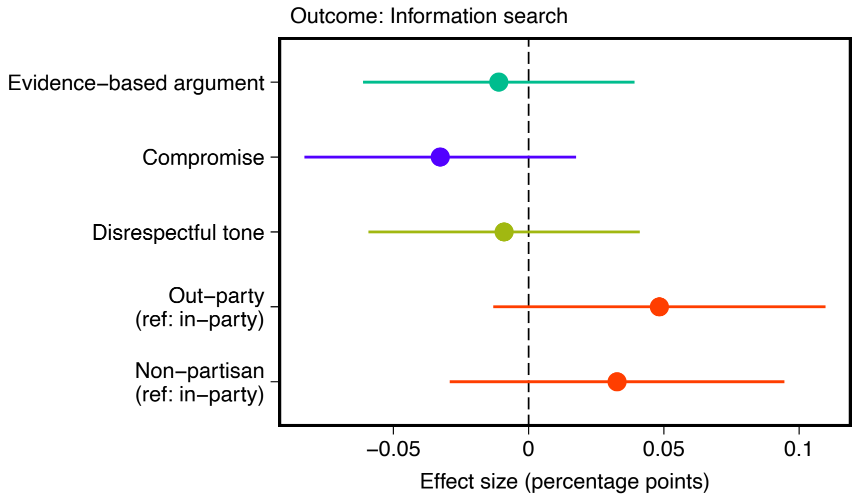

Figure S14: **United Kingdom: Effects of attributes of counter-argument on probability of clicking information link.** Estimated treatment effects with 95% confidence intervals from OLS regression model that includes all treatment variables.

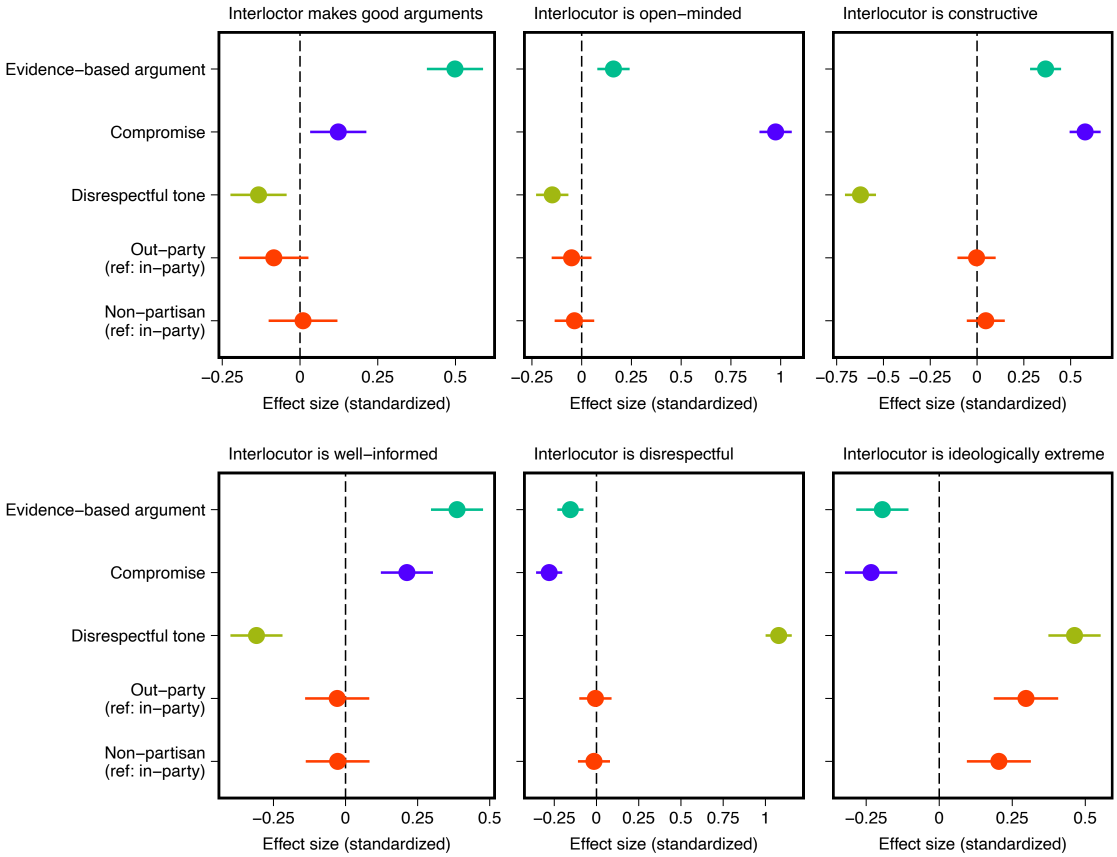

Figure S15: **United States: Effects of aspects of counter-argument on perceptions of the fictitious social media user.** Estimated treatment effects with 95% confidence intervals from OLS regression models that include all treatment variables.

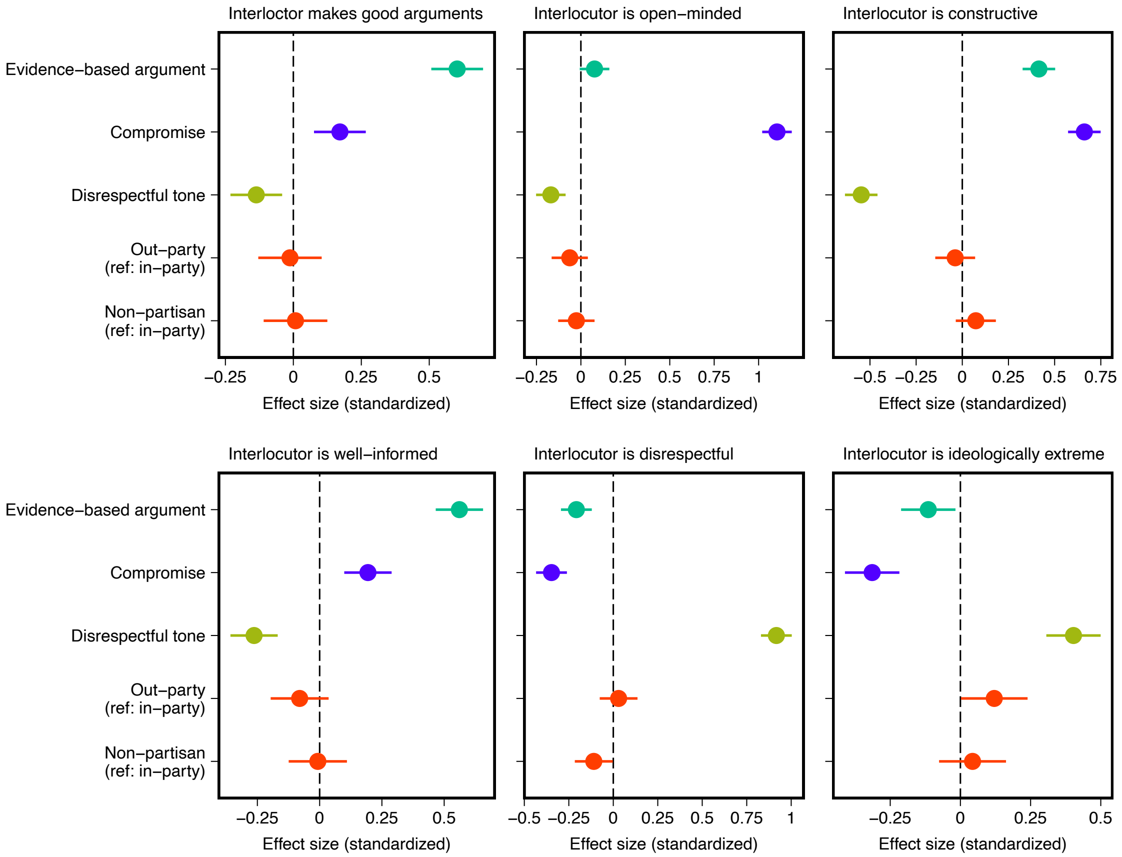

Figure S16: **United Kingdom: Effects of aspects of counter-argument on perceptions of the fictitious social media user.** Estimated treatment effects with 95% confidence intervals from OLS regression models that include all treatment variables.

S7. Quality of reply robustness checks

Analyses excluding issue positions that reference recent events

One potential consideration when using LLMs to develop counter-arguments on the fly is that the issue positions inputted by respondents may reference recent events that are outside of the LLMs' training data. This can potentially result in the LLM not being able to sufficiently address a given event or to write that such an event has not yet or did not occur. As a robustness check, we manually coded all issue positions from respondents for whether they reference an event that may have occurred after the end of the LLMs' training date cutoff. This represents roughly 9% of observations. Because the LLM may produce less accurate counter-arguments for these observations, we remove them from the data and rerun the main analysis. In Figure S17, we present the results for the "High-quality response" outcome (analogous to Figure 1 in the main article) for the full dataset, and for the subset of data that excludes any observation referring to an event outside of the LLMs' training data. As Figure S17 shows, the results are virtually identical whether or not these observations are included.

Analyses with alternative quality measures

Figure S18 shows results for the two alternative continuous outcome measures of response quality (counting quality aspect of the reply) along side the primary binary quality outcomes. The results are very similar, except that partisanship only matters when focusing on the binary high quality outcome.

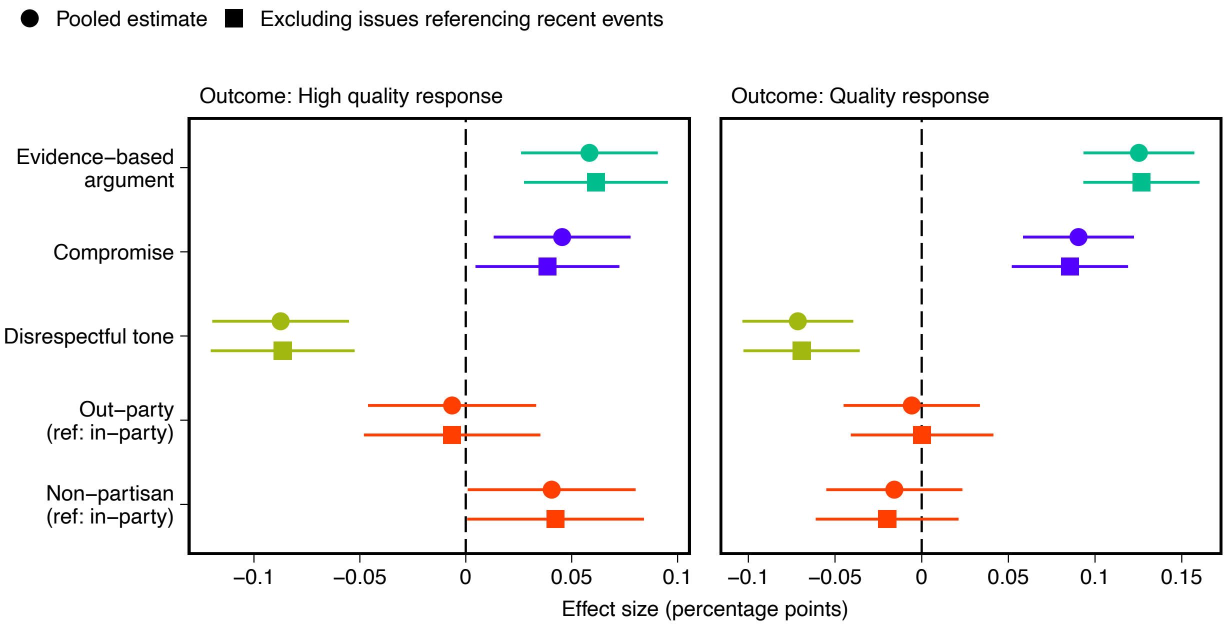

Figure S17: **Effects of attributes of counter-argument on probability of high-quality and quality reply (excluding issues referencing recent events).** Estimated treatment effects with 95% confidence intervals from OLS regression models that include all treatment variables.

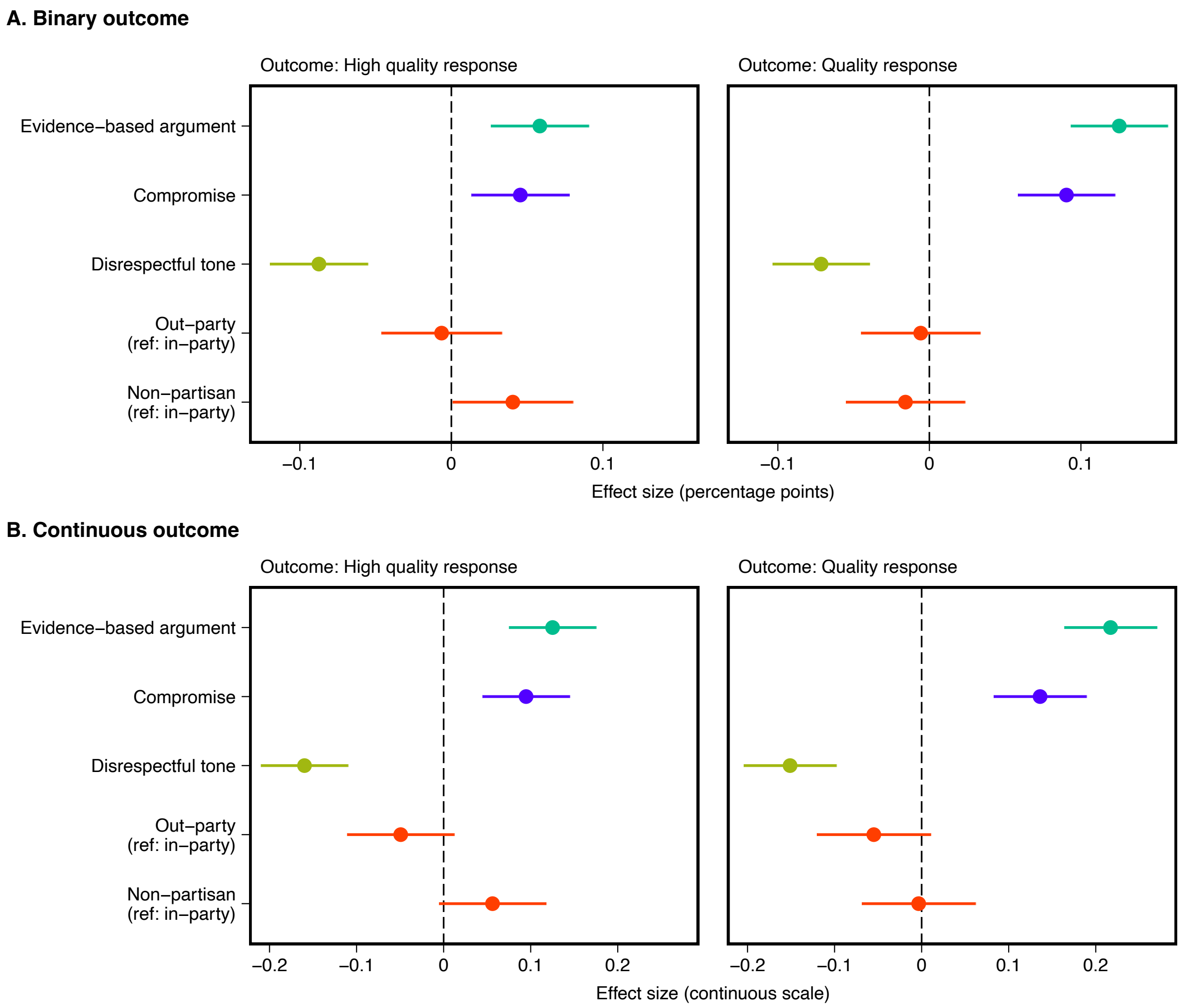

Figure S18: **Effects of attributes of counter-argument on probability of quality reply (alternative measures).** Estimated treatment effects with 95% confidence intervals from OLS regression models that include all treatment variables. "High quality response (binary outcome)" and "Quality response (binary outcome)" are the two response quality measures reported in Figure 1 in the main article. "High quality response (continuous outcome)" is the sum of quality aspects of the reply (not disrespectful, providing a qualified justification, not using partisan attacks, and showing willingness to compromise). 'Quality response (continuous outcome)" is similar to "High quality response (continuous outcome)" except using any justification instead of a qualified justification.

S8. Measurement of perceptions of the interlocutor

In the Results section concerning perceptions of the interlocutor, we combined, in an additive scale, perceptions of whether an argument is "reasonable" and whether it is "strong". In the first panel of Figure S19 we separate out the two components of these perceptions. As the figure shows, treatment effects for whether an interlocutor is perceived as making "strong" or "reasonable" arguments are in the same direction. Providing evidence-based arguments, indicating a willingness to compromise, and being disrespectful nevertheless have stronger effects on perceptions of whether an interlocutor makes "reasonable" arguments than on perceptions of "strong" arguments.

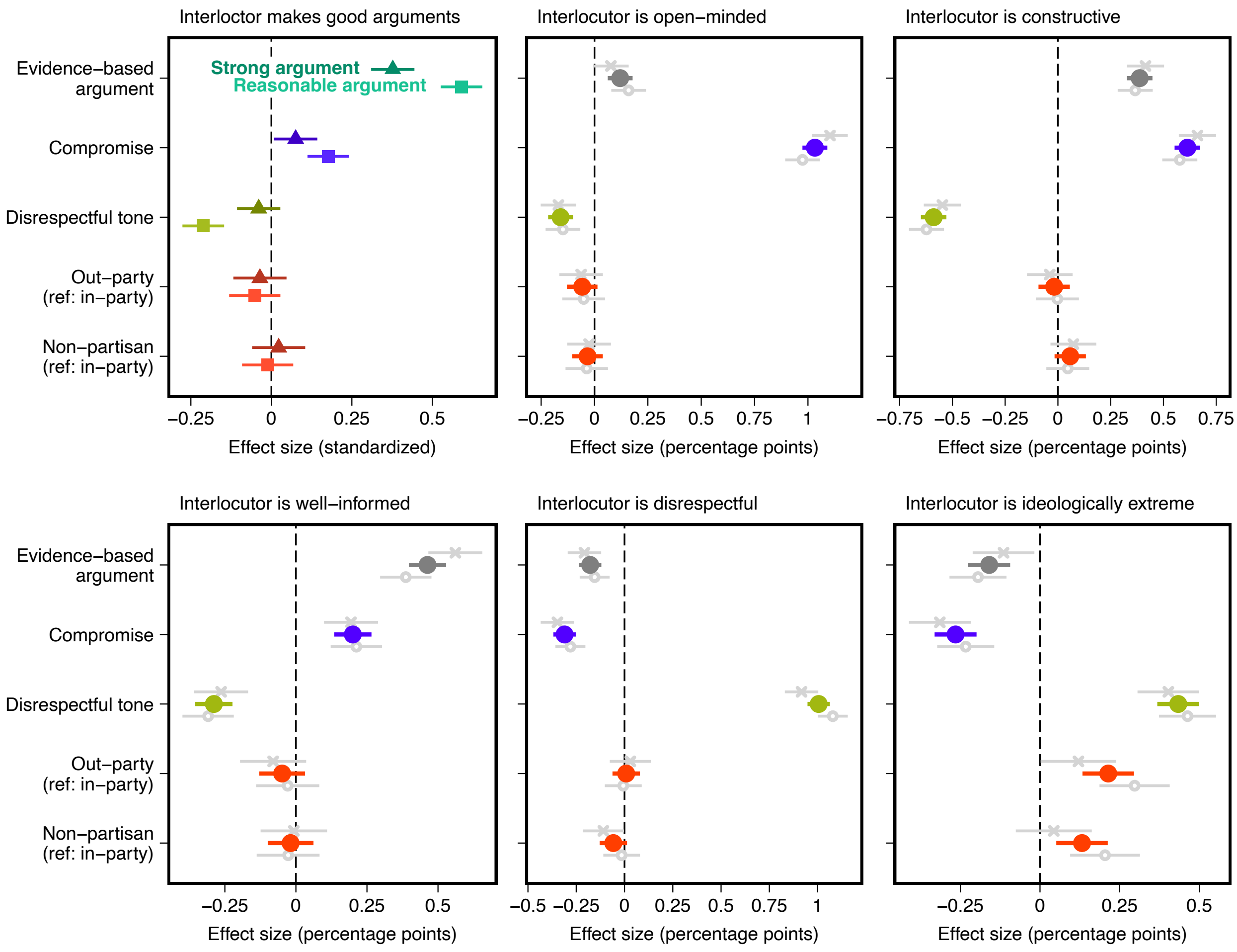

Figure S19: **Effects of attributes of counter-argument on perceptions of the interlocutor.** Estimated treatment effects with 95% confidence intervals from OLS regression models that include all treatment variables.

S9. Exploratory analyses

In this section, we present a number of exploratory analyses. Figure S20 tests the effect of the treatments on whether respondents make any mentions of partisanship. As the figure shows, using evidence-based arguments, signaling willingness to compromise, and not signaling one's own partisanship all reduce the probability of any mentions of partisanship. In Figure S21 we test whether the evidence-based condition, compromise treatment, and partisanship treatments differ in effect size if the response is disrespectful. It may be the case, for example, that being disrespectful minimizes the benefits of using evidence-based argumentation. As the figure shows, however, we find no evidence that the effects of these treatments are moderated by whether the response is also disrespectful.

In Figure S22 we test whether the disrespect treatment, evidence-based treatment, and compromise treatment differ in magnitude depending on whether the interlocutor is a member of an in-party, out-party, or signals no partisanship. In general, we find no strong evidence of differential treatment effects. The only significant difference is for the effect of evidence for interlocutors from the in-party and those who signal non-partisanship, with the evidence-based treatment statistically significantly stronger when the argument comes from a member of the in-party ($p = 0.02$). Whether people are more likely to engage in higher quality debate when evidence comes from a member of the in-party is an important avenue for future research.

Finally, in Figure S23 thru Figure S30 we test for heterogeneous effects by a number of respondent characteristics. As the figures show, treatment effects are generally similar across sub-groups.

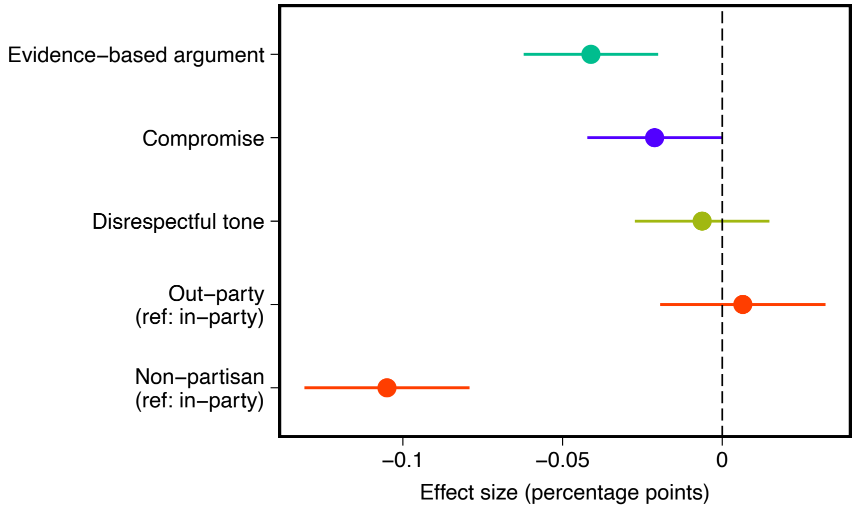

Figure S20: **Effects of attributes of counter-argument on probability of partisan reply.** Estimated treatment effects with 95% confidence intervals from OLS regression models that include all treatment variables. The outcome is a text-based measure of whether the reply contains any mentions of a party or politician.

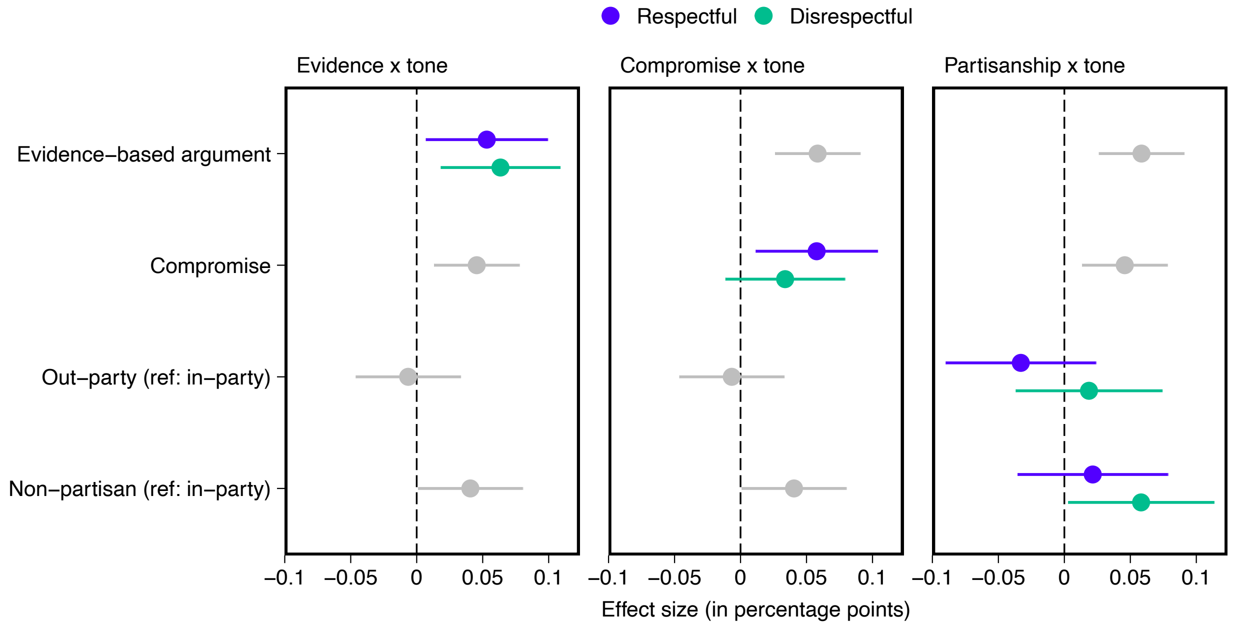

Figure S21: **Does a disrespectful tone reduce the effects of the other attributes of a counter-argument on the probability of a high-quality reply?** Estimated treatment effects with 95% confidence intervals from OLS regression models that include all treatment variables. The panel presents the effects of evidence-based arguments, signaling willingness to compromise, and partisanship, under the conditions of disrespect and respect. The magnitude of these treatment effects is not significantly different between the disrespect and respect conditions.

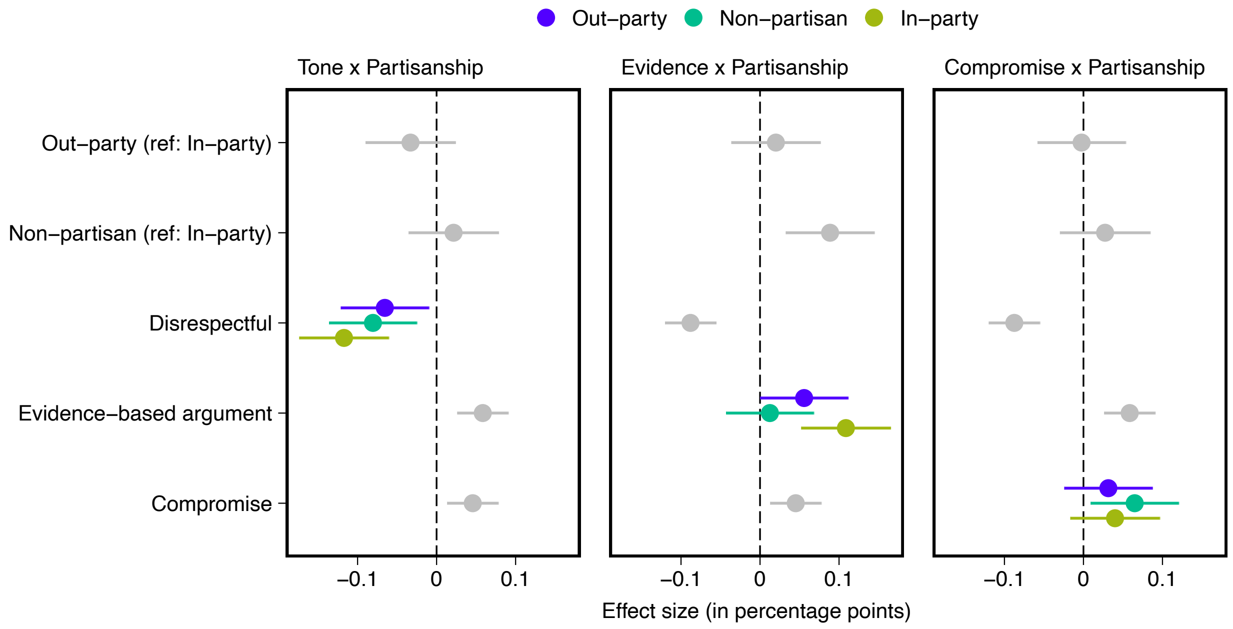

Figure S22: **Does the in-partisanship or out-partisanship of the interlocutor modify the effects of tone, argument type, and compromise?** Estimated treatment effects with 95% confidence intervals from OLS regression models that include all treatment variables. The panel presents the effects of evidence-based arguments, signaling willingness to compromise, and tone, under the condition that the interlocutor is member of the out-party or in-party, or non-partisanship.

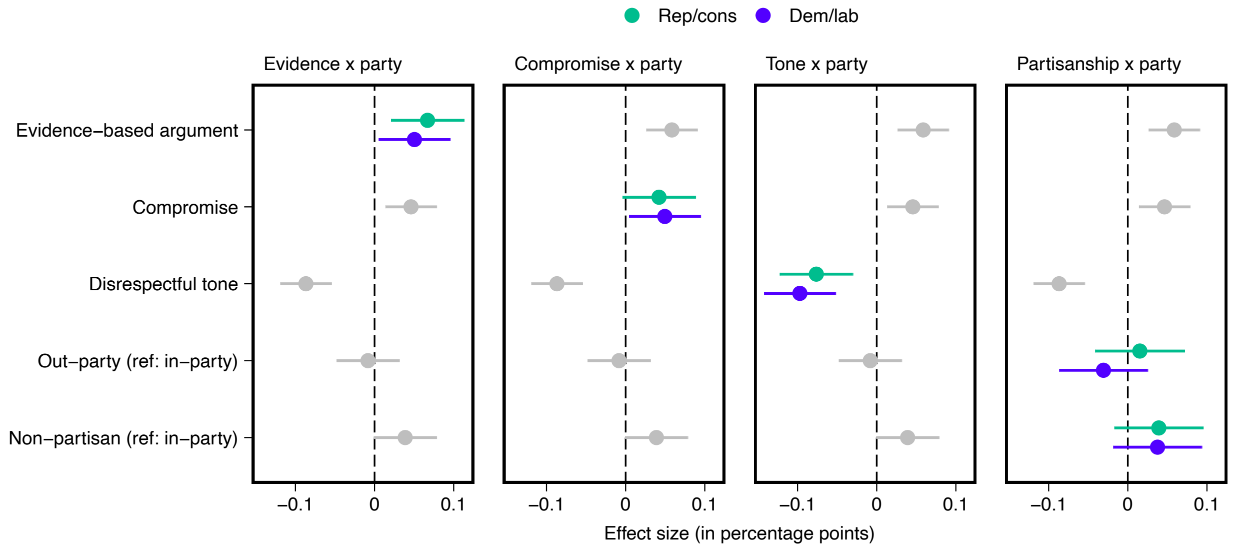

Figure S23: **Party asymmetry in treatment effects on the probability of a high-quality reply.** Estimated treatment effects with 95% confidence intervals from OLS regression models that include all treatment variables. Each panel shows the difference in the magnitude of the effect of a specific treatment when interacted with the party identification of the respondent (Republicans (US) and Conservatives (UK) vs. Democrats (US) and Labour supporters (UK)). None of the interactions are statistically significant.

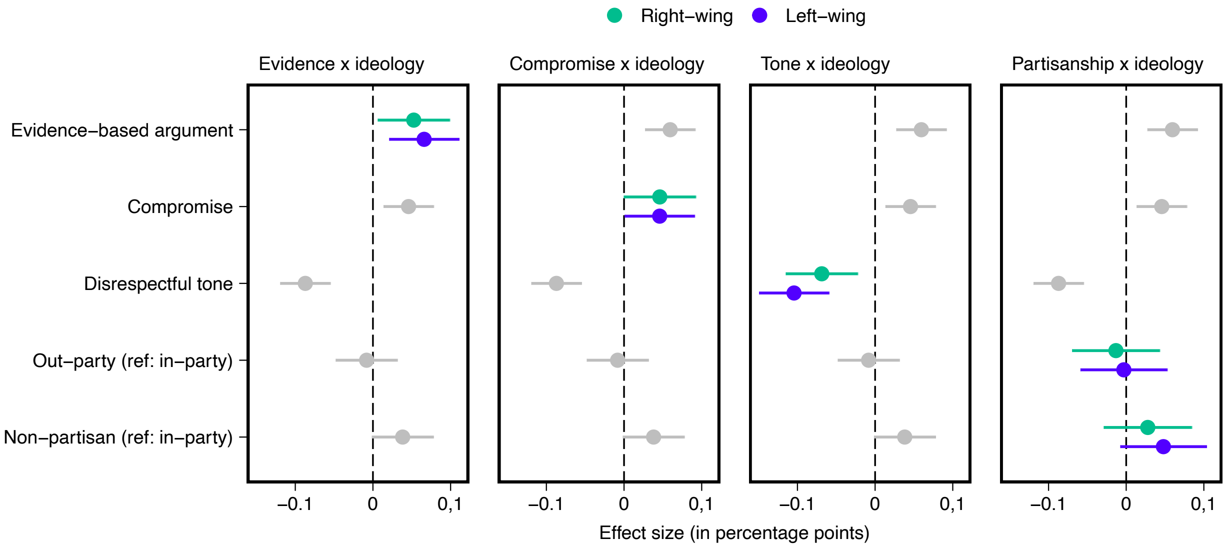

Figure S24: **Ideological asymmetry in effects of attributes of counter-argument on probability of high-quality reply.** Estimated treatment effects with 95% confidence intervals from OLS regression models that include all treatment variables. Each panel shows the difference in the magnitude of the effect of a specific treatment when interacted with the ideology of the respondent (right-wing vs. left-wing). None of the interactions are statistically significant.

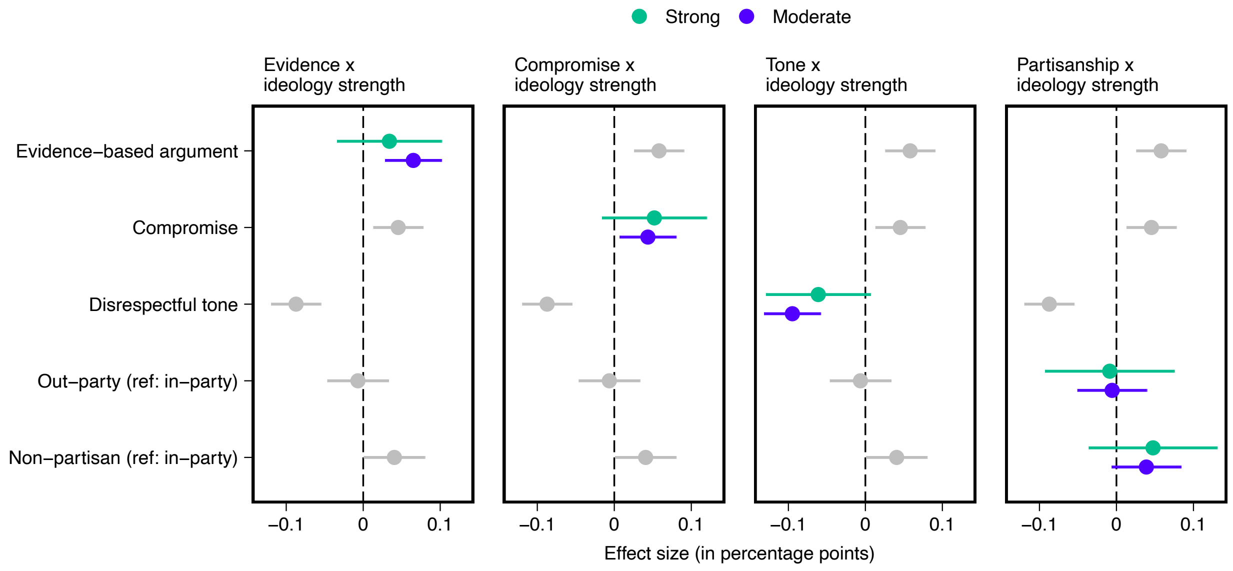

Figure S25: **Differential effects of attributes of counter-argument on probability of high-quality reply depending on ideological extremity.** Estimated treatment effects with 95% confidence intervals from OLS regression models that include all treatment variables. Each panel shows the difference in the magnitude of the effect of a specific treatment when interacted with the ideological strength of respondents ("Moderate" is slightly or somewhat left-wing/right-wing and "Strong" is very left-wing/right-wing). None of the interactions are statistically significant.

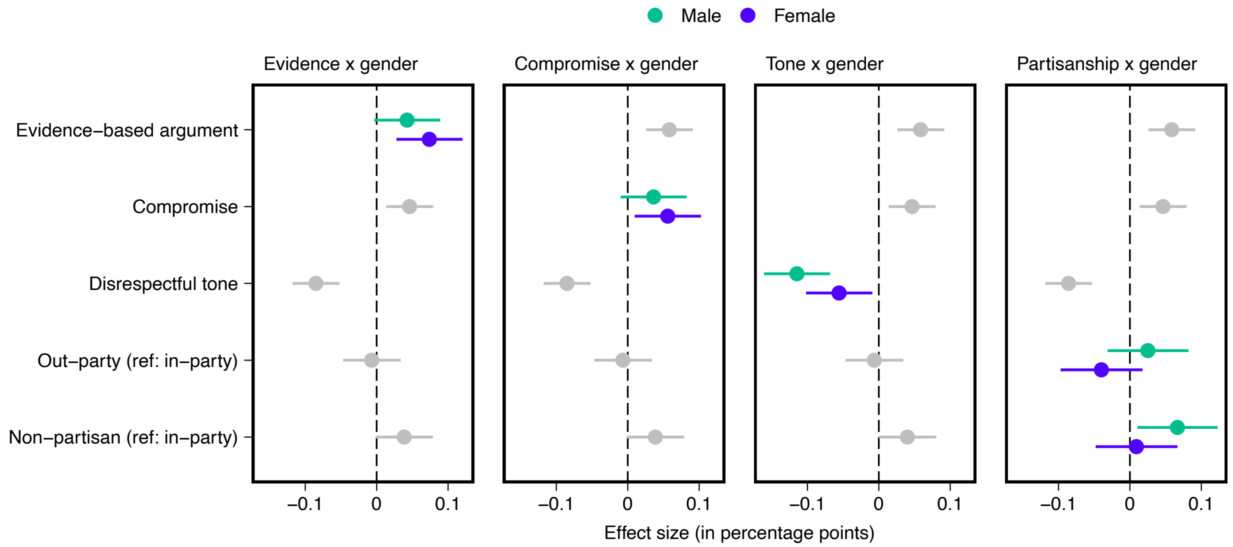

Figure S26: **Differential effects of attributes of counter-argument on probability of high-quality reply depending on gender.** Estimated treatment effects with 95% confidence intervals from OLS regression models that include all treatment variables. Each panel shows the difference in the magnitude of the effect of a specific treatment when interacted with the gender of the respondent. None of the interactions are statistically significant.

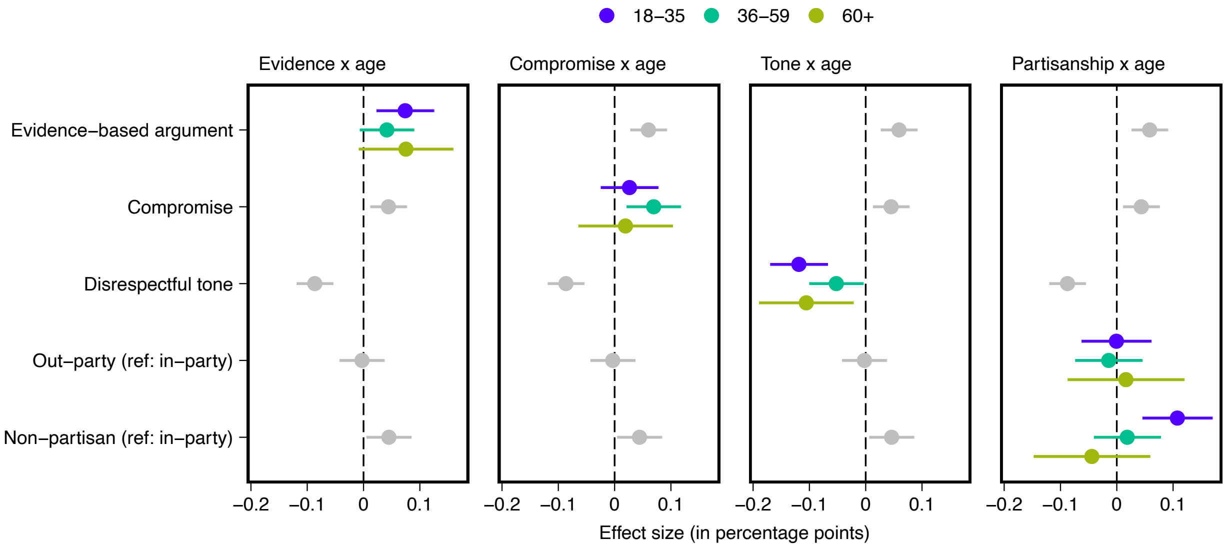

Figure S27: **Differential effects of attributes of counter-argument on probability of high-quality reply depending on age.** Estimated treatment effects with 95% confidence intervals from OLS regression models that include all treatment variables. Each panel shows the difference in the magnitude of the effect of a specific treatment when interacted with the age group of the respondent. The differences in the effect of the non-partisanship treatment between 18-35 years-olds, on the one hand, and 36-60 and 60+ years old, on the other hand, are statistically significant. All other differences are not statistically significant.

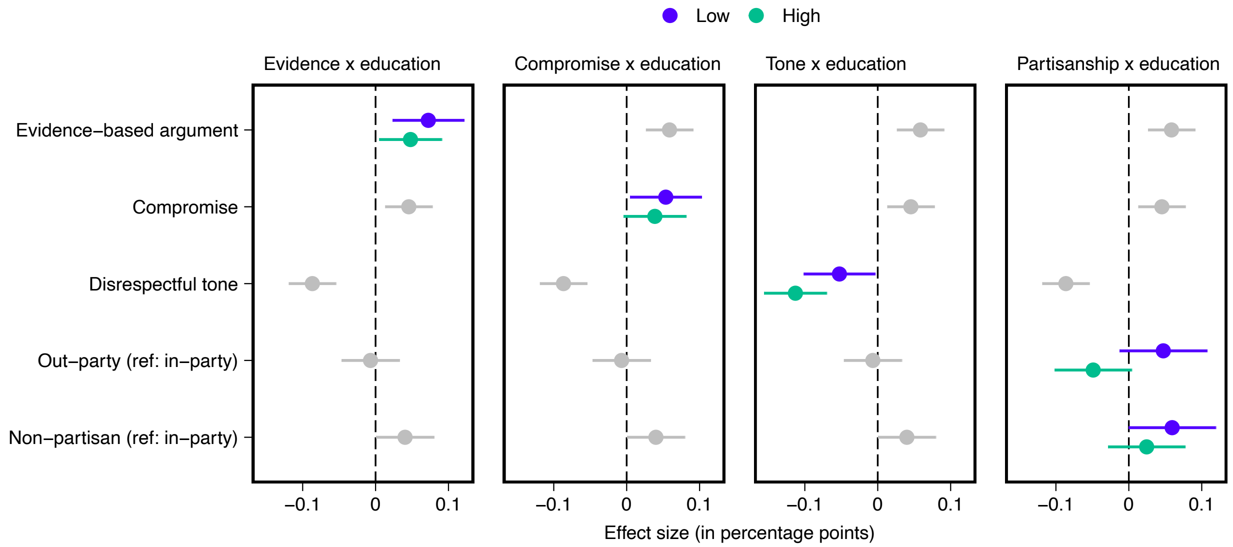

Figure S28: **Differential effects of attributes of counter-argument on probability of high-quality reply depending on education.** Estimated treatment effects with 95% confidence intervals from OLS regression models that include all treatment variables. Each panel shows the difference in the magnitude of the effect of a specific treatment when interacted with a respondent's level of education. Education is coded "High" if a respondent has an undergraduate or postgraduate degree, and "Low" otherwise. The difference in the effect of the out-party treatment between the two educational groups is statistically significant. All the other differences are not statistically significant.

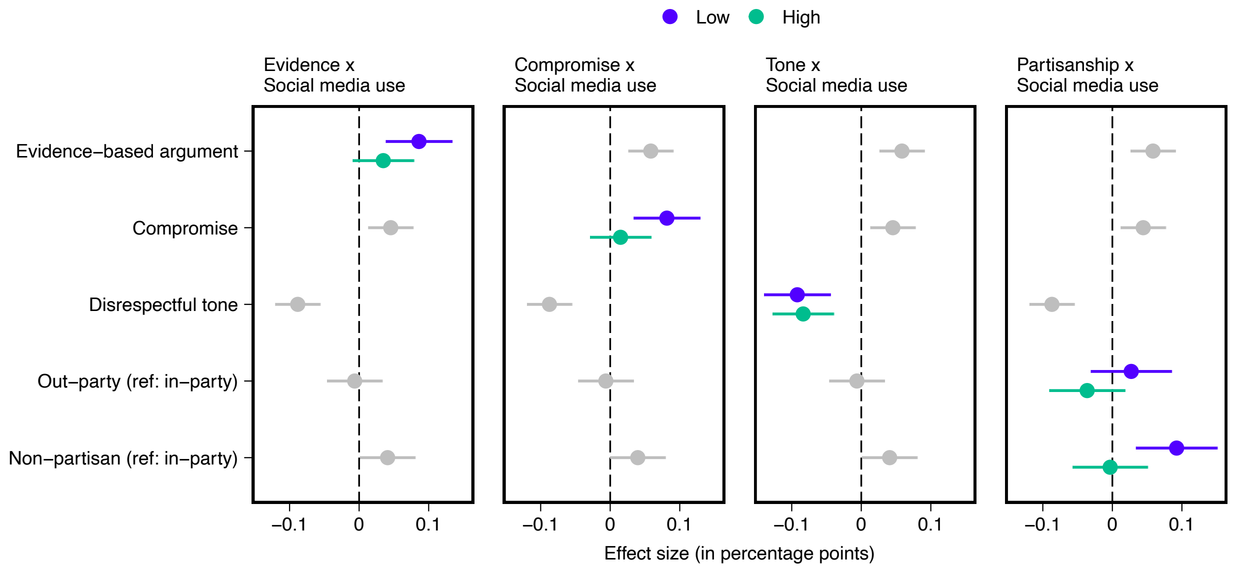

Figure S29: **Differential effects of attributes of counter-argument on probability of high-quality reply depending on social media (SoMe) engagement.** Estimated treatment effects with 95% confidence intervals from OLS regression models that include all treatment variables. Each panel shows the difference in the magnitude of the effect of a specific treatment when interacted with social media use of the respondent ("Low": < 0.5 hours per day; "High" ≥ 0.5 hours). The difference in the effects of the non-partisanship and compromise treatments between the low and high social media usage groups is statistically significant. All other differences are not statistically significant.

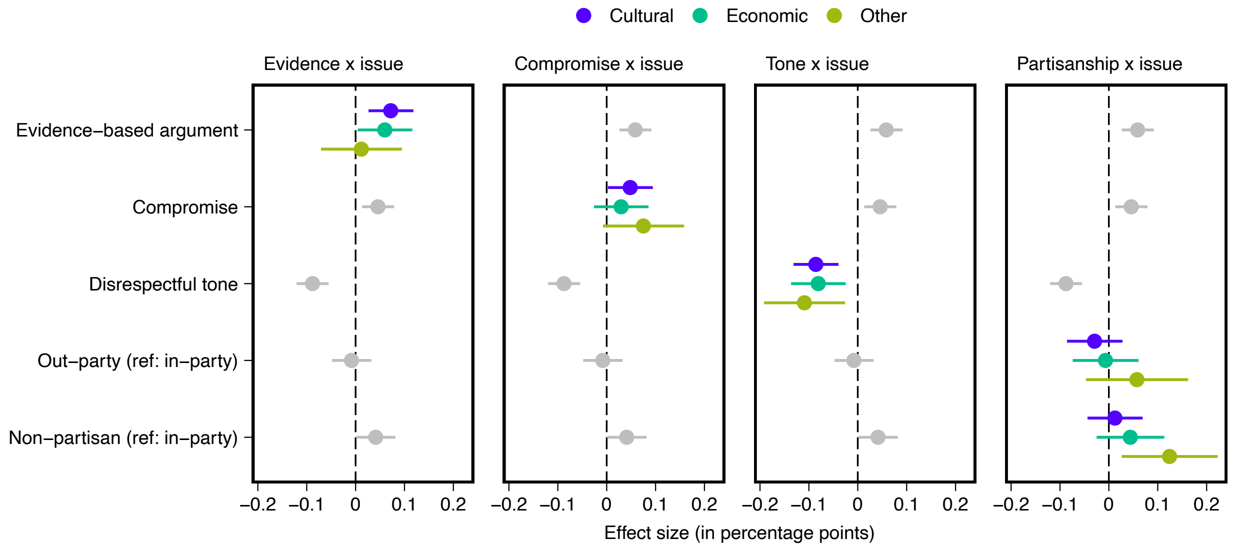

Figure S30: **Differential effects of attributes of counter-argument on probability of high-quality reply depending on type of issue (cultural/economic/other).** Estimated treatment effects with 95% confidence intervals from OLS regression models that include all treatment variables. Each panel shows the difference in the magnitude of the effect of a specific treatment when interacted with the type of issue a respondent discusses. The differences in the effect of the non-partisanship and out-party treatments between "Cultural" and "Other" issues are statistically significant. All other differences are not statistically significant.

S10. Regression tables for article figures

For each of the figures in the main article, we provide the analogous regression tables in Figure S13 (Figure 1 of main article), Figure S14 (Figure 2, top-left panel of main article), Figure S15 (Figure 2 top-right and bottom panels of main article), Figure S16 (Figure 3 of main article), Figure S17 (Figure 4 of main article), Figure S18 (Figure 5 of main article), and Figure S19 (Figure 6 of main article).

Table S13: **Figure 1 regression table: Effects of attributes of counter-argument on probability of high-quality and quality reply.**

	**High-quality response**			**Quality response**		
	**Pooled**	**UK**	**US**	**Pooled**	**UK**	**US**
Disrespectful tone	-0.09***	-0.06**	-0.12***	-0.07***	-0.06*	-0.09***
	(0.02)	(0.02)	(0.02)	(0.02)	(0.02)	(0.02)
Evidence-based argument	0.06***	0.07**	0.04	0.13***	0.15***	0.10***
	(0.02)	(0.02)	(0.02)	(0.02)	(0.02)	(0.02)
Compromise	0.05**	0.06*	0.03	0.09***	0.08***	0.10***
	(0.02)	(0.02)	(0.02)	(0.02)	(0.02)	(0.02)
Out-party (ref: in-party)	-0.01	-0.02	0.01	-0.01	-0.02	0.01
	(0.02)	(0.03)	(0.03)	(0.02)	(0.03)	(0.03)
Non-partisan (ref: in-party)	0.04*	0.01	0.07*	-0.02	-0.02	-0.01
	(0.02)	(0.03)	(0.03)	(0.02)	(0.03)	(0.03)
N	3303	1766	1537	3303	1766	1537

* $p < 0.05$, ** $p < 0.01$, *** $p < 0.001$

Table S14: **Figure 2 regression table (top-left panel): Effects of attributes of counter-argument on detailed discourse-level outcomes of the reply.**

	Provides qualified justification			Provides any justification		
	**Pooled**	**UK**	**US**	**Pooled**	**UK**	**US**
Disrespectful tone	0.01	0.00	0.01	0.02	0.03	0.00
	(0.01)	(0.02)	(0.02)	(0.02)	(0.02)	(0.02)
Evidence-based argument	0.10***	0.11***	0.10***	0.19***	0.20***	0.19***
	(0.01)	(0.02)	(0.02)	(0.02)	(0.02)	(0.02)
Compromise	0.01	0.00	0.02	0.05**	0.06*	0.05*
	(0.01)	(0.02)	(0.02)	(0.02)	(0.02)	(0.02)
Out-party (ref: in-party)	-0.00	0.00	-0.01	-0.01	-0.02	0.00
	(0.02)	(0.02)	(0.02)	(0.02)	(0.03)	(0.03)
Non-partisan (ref: in-party)	0.02	0.03	0.02	-0.04	-0.06	-0.02
	(0.02)	(0.02)	(0.02)	(0.02)	(0.03)	(0.03)
N	3303	1537	1766	3303	1537	1766

* p < 0.05, ** p < 0.01, *** p < 0.001

Table S15: **Figure 2 regression table (top-right panel and bottom panels): Effects of attributes of counter-argument on detailed discourse-level outcomes of the reply.**

	Compromise			Disrespectful tone			Partisan attack		
	**Pooled**	**UK**	**US**	**Pooled**	**UK**	**US**	**Pooled**	**UK**	**US**
Disrespectful tone	-0.10***	-0.14***	-0.07***	0.07***	0.05***	0.08***	-0.00	0.00	-0.01
	(0.01)	(0.02)	(0.02)	(0.01)	(0.01)	(0.01)	(0.01)	(0.01)	(0.01)
Evidence-based argument	-0.01	-0.03	0.01	-0.02*	-0.03*	-0.02	-0.01	-0.01	-0.01
	(0.01)	(0.02)	(0.02)	(0.01)	(0.01)	(0.01)	(0.01)	(0.01)	(0.01)
Compromise	0.04**	0.05*	0.04*	-0.03**	-0.04**	-0.02	-0.01	-0.01	-0.00
	(0.01)	(0.02)	(0.02)	(0.01)	(0.01)	(0.01)	(0.01)	(0.01)	(0.01)
Out-party (ref: in-party)	0.00	0.01	0.00	0.02	0.02	0.02	0.03***	0.02*	0.04**
	(0.02)	(0.03)	(0.02)	(0.01)	(0.02)	(0.02)	(0.01)	(0.01)	(0.01)
Non-partisan (ref: in-party)	0.03	0.06*	0.01	0.01	0.01	0.01	-0.01	-0.03*	0.00
	(0.02)	(0.03)	(0.02)	(0.01)	(0.02)	(0.02)	(0.01)	(0.01)	(0.01)
N	3303	1537	1766	3303	1537	1766	3303	1537	1766

* p < 0.05, ** p < 0.01, *** p < 0.001

Table S16: **Figure 3 regression table: Effects of attributes of counter-argument on openness to other beliefs.**

	Openness		
	**Pooled**	**UK**	**US**
Disrespectful tone	-0.10**	-0.12*	-0.07
	(0.04)	(0.05)	(0.05)
Evidence-based argument	0.12***	0.09	0.15**
	(0.04)	(0.05)	(0.05)
Compromise	0.01	0.02	0.02
	(0.04)	(0.05)	(0.05)
Out-party (ref: in-party)	-0.04	-0.06	-0.02
	(0.04)	(0.06)	(0.06)
Non-partisan (ref: in-party)	-0.02	-0.00	-0.04
	(0.04)	(0.06)	(0.06)
N	3231	1510	1721

* p < 0.05, ** p < 0.01, *** p < 0.001

Table S17: **Figure 4 regression table: Effects of attributes of counter-argument on affective and ideological polarization.**

	Affective polarization			Ideological polarization		
	**Pooled**	**UK**	**US**	**Pooled**	**UK**	**US**
Disrespectful tone	0.02	-0.00	0.04	0.02	-0.02	0.04
	(0.03)	(0.05)	(0.05)	(0.03)	(0.05)	(0.04)
Evidence-based argument	0.02	-0.02	0.06	0.01	0.02	0.00
	(0.03)	(0.05)	(0.05)	(0.03)	(0.05)	(0.04)
Compromise	-0.02	-0.01	-0.04	-0.00	-0.01	-0.00
	(0.03)	(0.05)	(0.05)	(0.04)	(0.05)	(0.04)
Out-party (ref: in-party)	0.09*	0.11	0.06	0.07	0.06	0.10
	(0.04)	(0.06)	(0.06)	(0.04)	(0.06)	(0.05)
Non-partisan (ref: in-party)	0.09*	0.15*	0.01	0.09*	0.08	0.11
	(0.04)	(0.06)	(0.06)	(0.04)	(0.06)	(0.05)
N	3275	1747	1528	3271	1743	1528

* p < 0.05, ** p < 0.01, *** p < 0.001

Table S18: **Figure 5 regression table: Effects of attributes of counter-argument on attitude strength and attitude certainty regarding the issue.**

	**Attitude strength**			**Attitude certainty**		
	**Pooled**	**UK**	**US**	**Pooled**	**UK**	**US**
Disrespectful tone	-0.03	0.03	-0.12*	0.01	0.04	-0.03
	(0.04)	(0.05)	(0.05)	(0.04)	(0.05)	(0.05)
Evidence-based argument	0.00	-0.01	0.01	-0.05	-0.06	-0.05
	(0.04)	(0.05)	(0.05)	(0.04)	(0.05)	(0.05)
Compromise	0.02	0.03	0.00	0.02	0.02	0.02
	(0.04)	(0.05)	(0.05)	(0.04)	(0.05)	(0.05)
Out-party (ref: in-party)	-0.02	0.01	-0.05	0.01	0.08	-0.07
	(0.04)	(0.06)	(0.06)	(0.04)	(0.06)	(0.06)
Non-partisan (ref: in-party)	0.03	0.09	-0.05	0.02	0.10	-0.07
	(0.04)	(0.06)	(0.06)	(0.04)	(0.06)	(0.06)
N	3230	1720	1510	3231	1721	1510

* p < 0.05, ** p < 0.01, *** p < 0.001

Table S19: **Figure 6 regression table: Effects of attributes of counter-argument on perceptions of the interlocutor.**

	**Makes good arguments**			**Open-minded**			Constructive			**Well-informed**			**Disrespectful**			**Ideologically extreme**		
	**Pooled**	**UK**	**US**	**Pooled**	**UK**	**US**	**Pooled**	**UK**	**US**	**Pooled**	**UK**	**US**	**Pooled**	**UK**	**US**	**Pooled**	**UK**	**US**
Disrespectful tone	-0.14***	-0.14**	-0.14**	-0.04	-0.06	-0.06	-0.21***	-0.18***	-0.18***	-0.16***	-0.17***	-0.17***	-0.59***	-0.55***	-0.55***	-0.29***	-0.26***	-0.26***
	(0.03)	(0.05)	(0.05)	(0.03)	(0.05)	(0.05)	(0.03)	(0.05)	(0.05)	(0.03)	(0.04)	(0.04)	(0.03)	(0.04)	(0.04)	(0.03)	(0.05)	(0.05)
Evidence-based argument	0.54***	0.60***	0.60***	0.38***	0.42***	0.42***	0.59***	0.64***	0.64***	0.12***	0.08	0.08	0.39***	0.41***	0.41***	0.46***	0.56***	0.56***
	(0.03)	(0.05)	(0.05)	(0.03)	(0.05)	(0.05)	(0.03)	(0.05)	(0.05)	(0.03)	(0.04)	(0.04)	(0.03)	(0.04)	(0.04)	(0.03)	(0.05)	(0.05)
Compromise	0.14***	0.17***	0.17***	0.08*	0.08	0.08	0.18***	0.23***	0.23***	1.03***	1.10***	1.10***	0.61***	0.66***	0.66***	0.20***	0.19***	0.19***
	(0.03)	(0.05)	(0.05)	(0.03)	(0.05)	(0.05)	(0.03)	(0.05)	(0.05)	(0.03)	(0.04)	(0.04)	(0.03)	(0.05)	(0.05)	(0.03)	(0.05)	(0.05)
Out-party (ref: in-party)	-0.05	-0.01	-0.01	-0.04	0.01	0.01	-0.05	-0.03	-0.03	-0.06	-0.06	-0.06	-0.02	-0.04	-0.04	-0.05	-0.08	-0.08
	(0.04)	(0.06)	(0.06)	(0.04)	(0.06)	(0.06)	(0.04)	(0.06)	(0.06)	(0.04)	(0.05)	(0.05)	(0.04)	(0.06)	(0.06)	(0.04)	(0.06)	(0.06)
Non-partisan (ref: in-party)	0.01	0.01	0.01	0.02	-0.00	-0.00	-0.01	0.02	0.02	-0.03	-0.03	-0.03	0.06	0.07	0.07	-0.02	-0.01	-0.01
	(0.04)	(0.06)	(0.06)	(0.04)	(0.06)	(0.06)	(0.04)	(0.06)	(0.06)	(0.04)	(0.05)	(0.05)	(0.04)	(0.06)	(0.06)	(0.04)	(0.06)	(0.06)
N	3280	1529	1529	3280	1529	1529	3280	1529	1529	3281	1530	1530	3280	1529	1529	3280	1529	1529

* p < 0.05, ** p < 0.01, *** p < 0.001
